# Innovative Multistage ML-QSAR Models for Malaria: From Data to Discovery

**DOI:** 10.1021/acsmedchemlett.4c00323

**Published:** 2024-07-18

**Authors:** Joyce V. B. Borba, Luis Carlos Salazar-Alvarez, Letícia Tiburcio Ferreira, Sabrina Silva-Mendonça, Meryck Felipe Brito da Silva, Igor H. Sanches, Leandro da Costa Clementino, Marcela Lucas Magalhães, Aline Rimoldi, Juliana Calit, Sofia Santana, Miguel Prudêncio, Pedro V. Cravo, Daniel Y. Bargieri, Gustavo C. Cassiano, Fabio T. M. Costa, Carolina Horta Andrade

**Affiliations:** † Laboratory of Tropical Diseases − Prof. Dr. Luiz Jacintho da Silva, Department of Genetics Evolution, Microbiology and Immunology. Institute of Biology, UNICAMP, 13083-970 Campinas, São Paulo Brazil; ‡ Laboratory for Molecular Modeling and Drug Design (LabMol), Faculty of Pharmacy, Federal University of Goias, Rua 240, qd. 87, Goiânia, Goiás 74605-170, Brazil; § Center for Excellence in Artificial Intelligence (CEIA), Institute of Informatics, Universidade Federal de Goiás, Goiânia, 74605-170, Goiás Brazil; ∥ Center for the Research and Advancement in Fragments and Molecular Targets (CRAFT), School of Pharmaceutical Sciences at Ribeirao Preto, University of São Paulo, Ribeirão Preto, São Paulo 14040-903, Brazil; ⊥ Department of Parasitology, Institute of Biomedical Sciences, University of São Paulo, 05508-000, São Paulo, São Paulo Brazil; ¶ Instituto de Medicina Molecular Jão Lobo Antunes, Faculdade de Medicina da Universidade de Lisboa, 1649-028 Lisboa, Portugal; ◆ Global Health and Tropical Medicine, Associate Laboratory in Translation and Innovation Towards Global Health, Instituto de Higiene e Medicina Tropical, Universidade NOVA de Lisboa, Rua da Junqueira 100, 1349-008 Lisbon, Portugal

**Keywords:** Artificial Intelligence, Liver stage, Sexual stage, Blood stage, Antimalarial, QSAR, Hits, *Plasmodium*

## Abstract

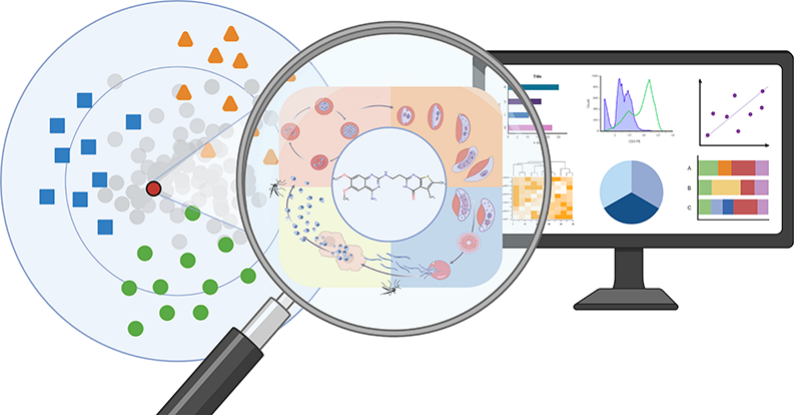

Malaria presents a significant challenge to global public health, with around 247 million cases estimated to occur annually worldwide. The growing resistance of *Plasmodium* parasites to existing therapies underscores the urgent need for new and innovative antimalarial drugs. This study leveraged artificial intelligence (AI) to tackle this complex challenge. We developed multistage Machine Learning Quantitative Structure–Activity Relationship (ML-QSAR) models to effectively analyze large datasets and predict the efficacy of chemical compounds against multiple life cycle stages of *Plasmodium* parasites. We then selected 16 compounds for experimental evaluation, six of which showed at least dual-stage inhibitory activity and one inhibited all life cycle stages tested. Moreover, explainable AI (XAI) analysis provided insights into critical molecular features influencing model predictions, thereby enhancing our understanding of compound interactions. This study not only empowers the development of advanced predictive AI models but also accelerates the identification and optimization of potential antiplasmodial compounds.

Malaria is a major disease in tropical and subtropical regions of the globe, caused by protozoan parasites of the genus *Plasmodium* and transmitted to humans by female *Anopheles* mosquitoes. In 2022, there were an estimated 200 million malaria cases and 600,000 deaths worldwide, with the disease remaining a significant public health issue, particularly in low-income countries.^1,2^ Among the six *Plasmodium* species that infect humans (*P. falciparum*, *P. vivax*, *P. malariae*, *P. ovale curtisi*, *P. ovale wallikeri*, and *P. knowlesi*), *P. vivax* and *P. falciparum* pose the greatest threat. *P. falciparum* dominates the global malaria burden, while *P. vivax* is the second most significant cause of disease, responsible for approximately 3.3% of global infections, including 75% of cases in the Americas and 50% in Southeast Asia.^3^

The *Plasmodium* life cycle involves a phase of sexual reproduction inside the mosquito vector, and phases of asexual multiplication in the liver and red blood cells of the mammalian host.^4^ Human infection begins when an infected *Anopheles* takes a blood meal, injecting *Plasmodium* sporozoites into the host’s skin. The sporozoites then reach the bloodstream and travel to the liver, where they infect hepatocytes and develop into schizonts. Schizonts rupture, releasing merozoites that cyclically invade and replicate inside red blood cells, initiating the symptomatic phase of the infection. Some merozoites develop into gametocytes, which may be taken up by mosquitoes during a subsequent blood meal. In the mosquito, gametocytes mature into gametes, fuse to form zygotes, and develop into oocysts, producing new sporozoites that migrate to the mosquito’s salivary glands, where they remain ready to infect another human.^2,4^

The primary strategy for malaria control involves disease treatment and management, which urgently requires the development of new antimalarials with novel mechanisms and no cross-resistance to existing drugs, along with multistage antiplasmodial activity.^5^ No single drug exists for clinical use against all stages of the parasite’s complex life cycle.^6^ While several first-line drugs target asexual blood-stage parasites,^7^ inhibiting liver schizonts and gametocytes is essential for preventing epidemics and protecting vulnerable populations, especially in the face of rising drug resistance.^8−10^

Artificial Intelligence (AI) has significantly advanced the field of antimalarial drug discovery by providing innovative methods to expedite and refine the identification of effective hits and leads.^11−13^ By analyzing large datasets containing information about chemical compounds and biological outcomes, machine learning (ML) algorithms can predict which compounds are most likely to be effective against the malaria parasite, allowing researchers to focus on the most promising leads.^11−14^ This approach not only accelerates the drug discovery process but also reduces costs and increases the likelihood of finding effective therapies. Here, we developed a multistage ML-Quantitative Structure–Activity Relationship (ML-QSAR) to identify new hits with multi-stage antiplasmodial activity. To this end, we analyzed large datasets and predicted the efficacy of chemical compounds against multiple life cycle stages of *Plasmodium* parasites. We then selected 16 compounds for experimental evaluation, six of which demonstrated dual- or triple-stage inhibitory activity. Notably, one compound inhibited over 50% across all stages tested (liver stage, asexual blood stage, gametocytes, and ookinetes). Additionally, explainable AI (XAI) analysis revealed key molecular features that influence model predictions, improving our understanding of compound interactions. This research underscores AI’s crucial role in accelerating the discovery and optimization of potential antimalarial therapies.

We compiled data from multiple publicly available databases, an *in house* database (ookinetes dataset), and the literature. Detailed information on the datasets is available at the GitHub repository. They include experimental evaluation on the inhibition of different life stages *of Plasmodium spp*.: (i) asexual blood stages of chloroquine-sensitive *P. falciparum* 3D7 strain (ABS-3D7 dataset); (ii) asexual blood stages of multidrug resistant *P. falciparum* W2 strain (ABS-W2 dataset); (iii) sexual stages in fertilization model of inhibition of *P. berghei* ookinetes formation (ookinetes dataset); (iv) *P. falciparum* gametocytes stages (gametocytes dataset); and (v) *P. berghei* hepatic schizonts stages (liver schizonts dataset). For a thorough comprehension of the datasets and access to carefully curated collections, refer to the computational procedures in the Supporting Information.

After data curation, the ABS-3D7 dataset included 35,943 compounds (15,208 active and 20,735 inactive), the ABS-W2 dataset had 6,724 compounds (3,362 active and 3,262 inactive), the ookinetes dataset included 1,562 compounds (293 active and 1,269 inactive), the gametocytes dataset had 558 compounds (166 active and 392 inactive), and the liver schizonts dataset included 1890 compounds (523 actives and 666 inactive). Following curation, the datasets were split into modeling (80%) and external (20%) subdatasets (Table 1).

**Table 1:** General Information about the Datasets Used for Model Building

Dataset	Active	Inactive	Total	Active/inactive proportion	Activity Threshold
**ABS-3D7**	15,208	20,735	35,943	1:1.4	1 μM
Modeling	12,166	16,588	28,754	1:1.4	
External	3,042	4,147	7,189	1:1.4	
**ABS-W2**	3,362	3,362	6,724	1:1	1 μM
Modeling	2,690	2,690	5,380	1:1	
External	672	672	1,344	1:1	
**Ookinetes**	293	1,269	1,562	1:4.3	2 μM
Modeling	234	1,015	1,249	1:4.3	
External	59	254	313	1:4.3	
**Gametocytes**	166	392	558	1:2.4	1 μM
Modeling	133	314	447	1:2.4	
External	33	78	111	1:2.4	
**Liver schizonts**	523	666	1,189	1:1.2	1 μM
Modeling	418	533	951	1:1.2	
External	105	133	238	1:1.2	

The chemical space of the datasets was visualized with a *t*-distributed stochastic neighbor embedding (*t*-SNE) analysis^15^ for dimension reduction to assess the diversity of collected compounds. For each dataset, Murcko scaffolds^16^ were calculated, compounds with the same scaffold were grouped, and their activity within each group was averaged. The scaffolds were represented by Extended Connectivity Fingerprints (ECFP4) generated by RDKit as input to *t*-SNE. The chemical space analysis is shown in Figure 1, showcasing a broad distribution of scaffolds, underscoring the extensive diversity within the collected molecules. Notably, all datasets demonstrate a substantial percentage of scaffolds, with the majority being unique, i.e., each scaffold being represented by a single molecule in its corresponding dataset. This scaffold variability demonstrates that these datasets contain a wide variety of compounds. Such diversity is crucial for developing models that can predict properties across a broad spectrum of molecules, thereby enhancing their applicability.

**Figure 1:**
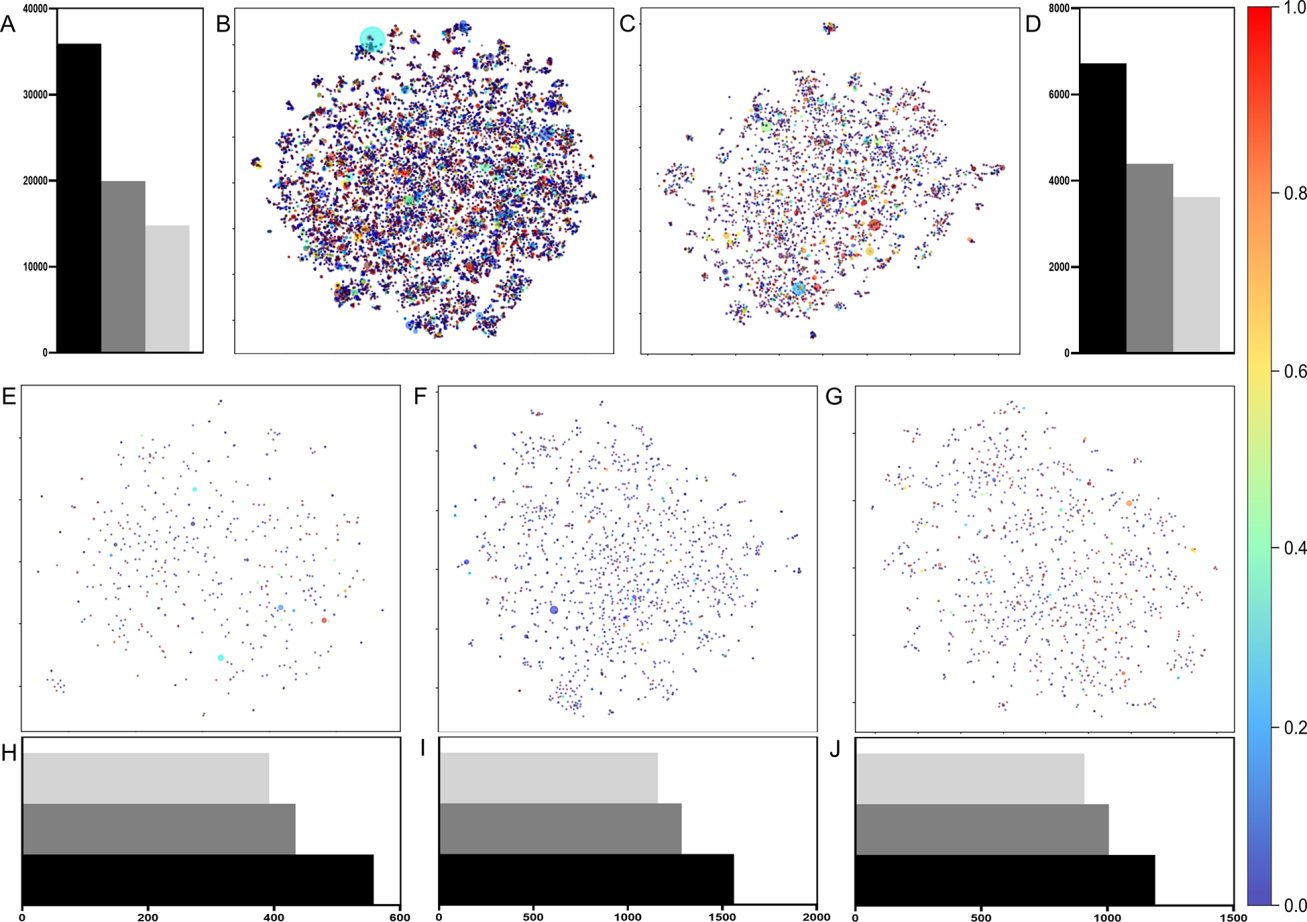
Data scaffold analysis using Murcko scaffolds and ECFP4 descriptors represented in a 2048-bit array. *t*-SNE was applied to reduce the descriptor dimensions to two. Panels (A, D, H, I, J) show scaffold group distributions within each dataset (ABS-3D7 in A, ABS-W2 in D, gametocytes in H, ookinetes in I, and liver schizonts in J), including counts of unique scaffolds associated with single molecules. Black bars indicate the number of compounds, dark gray bars depict total scaffold occurrences, and light gray bars represent unique scaffold counts. Panels (B, C, E, F, G) display t-SNE projections of the chemical space for ABS-3D7 (B), ABS-W2 (C), gametocytes (E), ookinetes (F), and liver schizonts (G) datasets. Circle sizes correspond to scaffold frequencies in each dataset, with larger circles indicating higher occurrence. Circle colors denote the mean activity of molecules associated with each scaffold, transitioning from red (0–all compounds with that scaffold are inactive) to blue (1–all compounds with that scaffold are active).

Figure S1 (Supporting Information) includes the most frequent scaffolds and associated activity probabilities for each dataset. For example, in the ABS-3D7 dataset, 19,943 distinct scaffolds are observed, with 14,807 appearing in only one representative molecule (Figure 1A). The three most prominent scaffold clusters within the ABS-3D7 dataset exhibit varying activity probabilities. Molecules containing an aminoquinoline scaffold are highly likely to be active, as evidenced by the presence of this core in prominent drugs such as chloroquine, amodiaquine, and piperaquine. In contrast, benzyl benzamide exhibits a lower probability of activity, and diphenylurea presents uncertain activity. This detailed analysis provides insights into the unique scaffold landscape identified and its correlation with molecular activity in the respective datasets. The ABS-W2 dataset contains 4,393 different scaffolds, 3,622 of which appeared in a single representative molecule (Figure 1D); the gametocytes dataset contains 434 different scaffolds, 392 of which appeared in a single representative molecule (Figure 1H); the ookinetes dataset contains 1,284 different scaffolds, 1,159 of which appeared in a single representative molecule (Figure 1I); and the liver schizonts dataset contains 1,005 different scaffolds, 908 of which appeared in a single representative molecule (Figure 1J).

ML-QSAR models were generated and validated using the described datasets to distinguish active from inactive compounds in the different life stages of *Plasmodium*. Overall, 24 models were built for each life stage through the combination of tree machine learning algorithms (Random Forest, Support Vector Machine, and Light Gradient Boosting Machine) with four types of molecular descriptors: MACCS, ECFP, FCFP and Mordred (radius 2: ECFP4, FCFP4; radius 4: ECFP8, FCFP8; hybrids: MACCS_Mordred, ECFP4_Mordred, and FCFP4_Mordred). The statistics results for the best models are available in Figure 2, and all generated models and their statistics are available at the GitHub repository.

**Figure 2:**
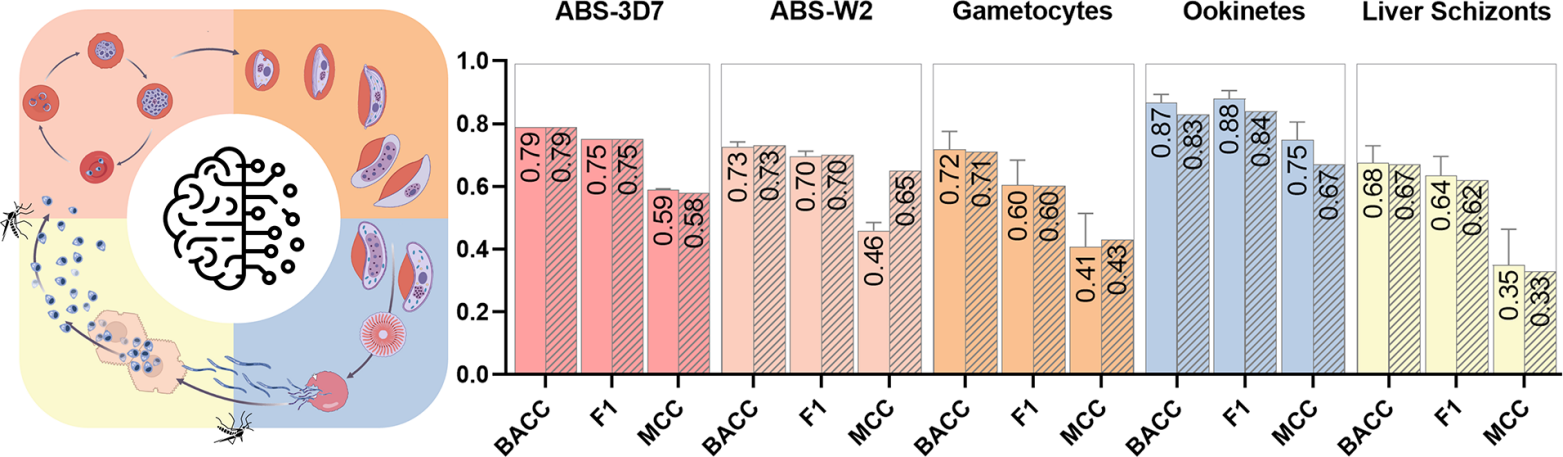
Statistical metrics for *5-fold* cross-validation (solid bars–mean and standard deviation) and external set validation (hatched bars) of the top performing ML-QSAR models developed for ABS-3D7 (dark pink), ABS-W2 (light pink), gametocytes (orange), ookinetes (blue), and liver schizonts (yellow) datasets. BACC: Balanced Accuracy; F1 score: harmonic mean of precision and recall; MCC: Mathew’s Correlation Coefficient.

The generated models were calibrated by altering the probability threshold (standard value = 50%) for class labeling.^17^ The probability estimation represents an important parameter for confidence evaluation in ML-QSAR model building. Compounds with estimated probabilities >50% are usually labeled as active, while compounds with <50% are labeled as inactive. However, the ML-QSAR models built with unbalanced data usually give poor probabilities for the minority class. Interstingly, even when the general performance is satisfactory, the model struggles to distinguish between classes, and the reliability of these predictions is low.^18,19^ Hence, the threshold of 50% might not represent an adequate cutoff for labeling compounds as either active or inactive. The statistical results for the calibrated models built are available in the GitHub repository.

The calibration approach significantly enhanced the statistical performance of the ABS-3D7, gametocyte, and liver schizont models (Figure 2). For the ABS-3D7 dataset, employing ECFP4 descriptors with the RF algorithm and a probability threshold of 39 yielded the best internal (Balanced Accuracy, BACC = 0.79, Sensitivity, SE = 0.71, and Specificity, SP = 0.86) and external (BACC = 0.79, SE = 0.83, and SP = 0.74) results. In the case of the gametocytes dataset, the hybrid descriptor combining ECFP4 and Mordred, along with the RF algorithm and a probability threshold of 47, delivered excellent internal (BACC = 0.72, SE = 0.72, and SP = 0.72) and external (BACC = 0.71, SE = 0.61, and SP = 0.82) performance metrics. Similarly, for the liver schizont dataset, employing FCFP4 descriptors with the RF algorithm and a probability threshold of 43 achieved robust internal (BACC = 0.68, SE = 0.67, and SP = 0.70) and external (BACC = 0.67, SE = 0.64, and SP = 0.71) results (Figure 2).

On the other hand, the calibration approach did not result in apparent improvements in the statistical performances of the models for the ABS-W2 and ookinetes datasets. The calibration thresholds calculated for these models were close to 50%, so they were kept at the standard threshold. Thus, the best model for the ABS-W2 dataset used the ECFP4 combined with RF algorithm and probability threshold of 50, and showed good internal (BACC = 0.73, SE = 0.64, and SP = 0.81) and external (BACC = 0.73, SE = 0.65, and SP = 0.81) statistic performances. The best model for the ookinetes dataset used the MACCS descriptors combined with RF algorithm and probability threshold of 50 and showed good internal (BACC = 0.87, SE = 0.95, and SP = 0.79) and external (BACC = 0.83, SE = 0.92, and SP = 0.75) statistic performances.

SHAP (Shapley Additive exPlanations)^20,21^ values were computed to assess the importance of the features of our ML-QSAR models. This analysis seeks to elucidate the specific features that exhibit the most significant contributions toward predictions within our multistage models (Figure 3). The summation of positive and negative contributions, incorporating the model’s base value (commonly referred to as the expected value, representing the average structural variability within the training dataset), yields a probability associated with a class label (in this context, the probability of antimalarial activity). The extent and directionality of these contributions, as captured by Shapley values, may vary for different compounds. Notably, Shapley Values analysis quantifies the impact of features absent in each compound on its prediction. This capability is paramount, as the absence of specific features can also be pivotal in shaping a prediction. Unfortunately, for the gametocytes model, Shapley values did not reveal distinct contributions for different features. This might have resulted from this model having hybrid descriptors (Mordred and ECFP4) that are scaled differently, causing their Shapley values to also be differentially scaled. For this reason, we decided not to use this methodology for the gametocytes model’s explainability.

**Figure 3:**
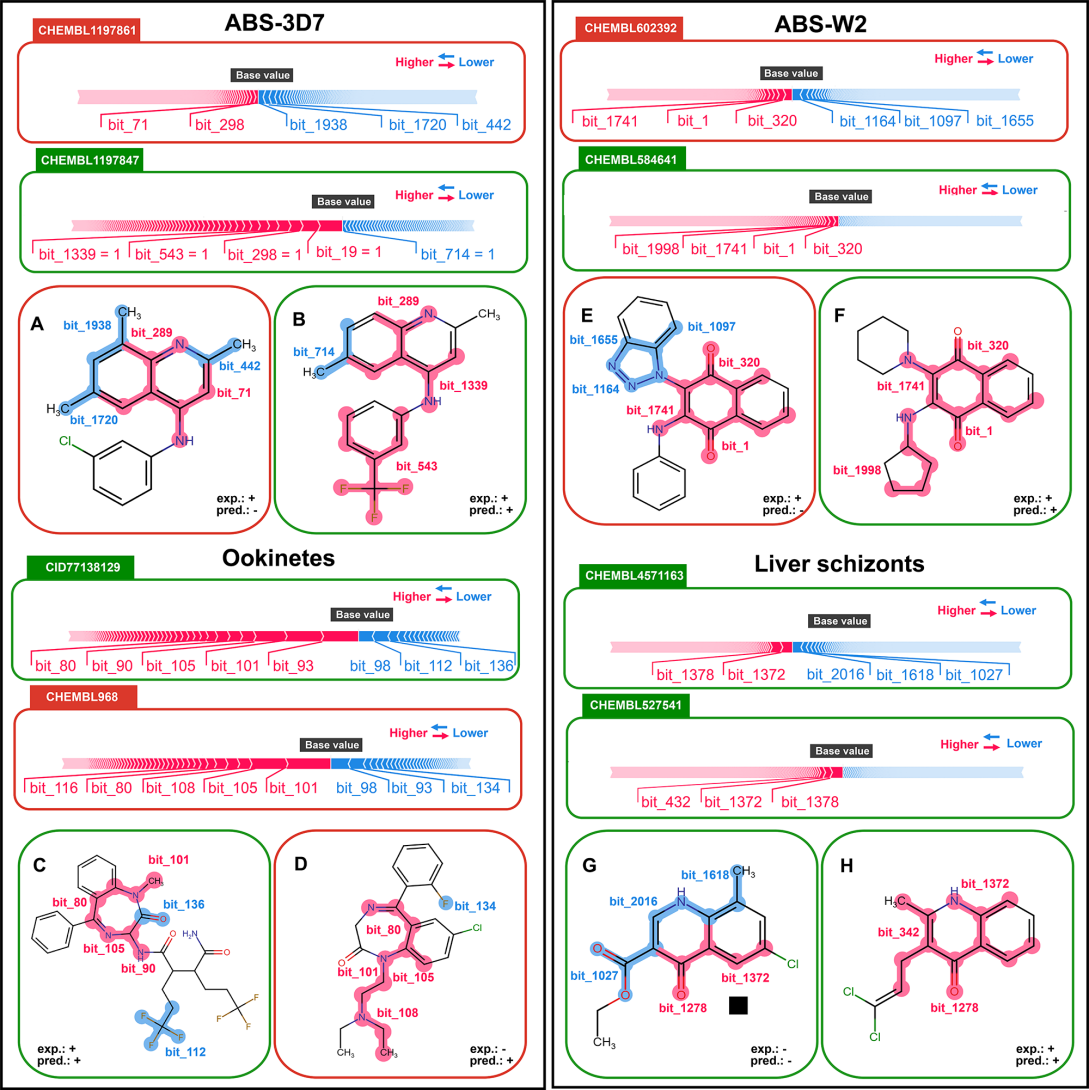
Local SHAP interpretation of the models on external set compounds is structurally similar but diverged in their predicted or experimentally observed outcomes. Compounds indicated inside green squares were correctly predicted, whereas those located inside red squares indicate inaccuracies in prediction. Red-highlighted SHAP fragments signify a beneficial effect on the model’s prediction, while blue-highlighted fragments signify a detrimental influence on the model’s prediction. exp. = experimental assignment of compound; pred = predicted assignment of compound; + = active; - = inactive.

We selected compounds from the external sets that were structurally similar but diverged in their predicted or experimentally observed outcomes. By examining these SHAP values, we aimed to understand which features significantly influenced the model’s decisions, particularly in cases where predictions conflicted with expectations or experimental results.

For the ABS-3D7 model (Figure 3A), we conducted a detailed analysis using two compounds, CHEMBL1197861 and CHEMBL119847, as a case study. Compound CHEMBL1197861, despite showing experimental activity against the ABS-3D7 strain, was predicted as inactive by the model. In contrast, CHEMBL119847 exhibited experimental activity consistent with the model’s prediction. Both compounds share an aminoquinoline scaffold, but differ in their substituents. In the SHAP analysis of CHEMBL1197861, it was revealed that methyl substituents exerted a negative influence on the predictions. Previous SAR studies have shown that electron-withdrawing substituents, such as chloride and bromide, at the seventh position of the quinoline ring enhance antiplasmodial activity.^22^ This aligns with the SHAP predictions, as methyl is an electron-donating group, explaining the negative influence on the prediction (depicted by bit_1720). Additionally, a chloroquine analog with a methyl substituent at the second position was found to be less potent against *P. falciparum* compared to chloroquine.^23^ The compound CHEMBL1197861 also has a methyl substituent at the second position (bit_442), which exerted a negative influence on prediction. Conversely, the presence of benzotrifluoride in CHEMBL119847 positively contributed to its predicted activity.

Compounds CHEMBL602392 and CHEMBL584641 were selected for the analysis of the ABS-W2 model (Figure 3B). CHEMBL602392 was predicted as inactive despite showing activity against the ABS-W2 strain. On the other hand, CHEMBL584641 was predicted as active and is experimentally active against the ABS-W2 strain. Both compounds share a naphthoquinone core, a significant factor contributing to a positive prediction in their SHAP analyses. However, the triazole ring negatively impacts the prediction of CHEMBL602392, as indicated by bits 1164, 1097, and 1655. Contrariwise, the presence of cyclopentylamine in CHEMBL584641 positively influences its predictive value, as indicated by bit 1998.

In the ookinetes model investigation (Figure 3C), we assessed compounds CID77138129 and CHEMBL968. CID77138129, which was experimentally active and correctly predicted as such, was compared to CHEMBL968, which was experimentally inactive and wrongly predicted as active. The benzodiazepine ring significantly contributes to the positive predictions of both compounds. However, it is worth noting that the long-chain substituent (bit 108) in compound CHEMBL968 could have also influenced the incorrect positive prediction.

Finally, compounds CHEMBL451163 and CHEMBL527541 were selected for the liver schizont model investigation (Figure 3D). Despite sharing the same core structure, both compounds exhibit opposing experimental activities in the liver schizonts stage. However, it is noteworthy that they were accurately predicted by the model. The benzodiazepine ring significantly contributes to the positive predictions of both compounds. Importantly, the presence of the carboxyl group (bit 1027) in compound CHEMBL451163 negatively contributed to the model’s prediction.

After the feature importance of our models was assessed, a virtual screening (VS) was conducted following the steps presented in Figure 4. We began by downloading and preparing the ChemBridge database version 2020, which contained 1,229,342 compounds. The database was then filtered using the five ML-QSAR models in the following sequence: ABS-3D7, ABS-W2, ookinetes, gametocytes, and liver schizonts stages. At each step, only compounds predicted to be active and within the applicability domain were retained. Initially, the ABS-3D7 model retained 142,020 compounds. The ABS-W2 model then predicted 13,382 of these compounds as active. Next, the ookinetes model retained 10,777 compounds, which were further filtered by the gametocytes model, resulting in 517 compounds. Finally, the liver schizonts model retained 228 compounds. In the end, only drug-like compounds were kept for further analysis. A literature search was conducted for the 61 remaining compounds, leading to the exclusion of eight compounds that had previously been tested against *Plasmodium spp*. parasites. The remaining compounds were then grouped into clusters based on structural similarity. After a thorough medicinal chemistry inspection of each cluster, 16 compounds were selected for experimental evaluation (Figure 4).

**Figure 4:**
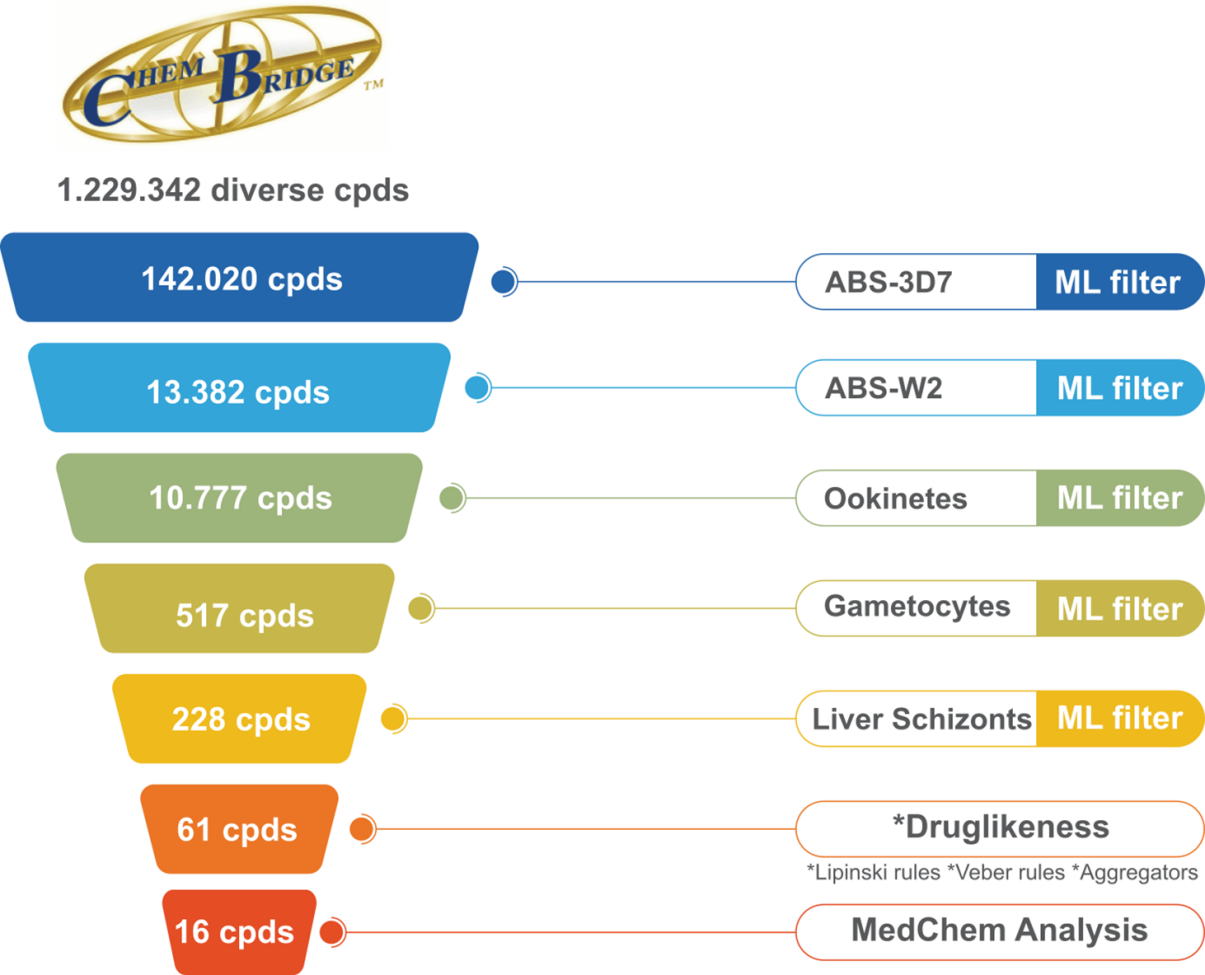
Virtual screening workflow based on the five ML-QSAR models for the identification of active compounds against multiple stages of *Plasmodium* parasites.

The ability of the 16 selected compounds to inhibit the asexual blood-stage of *P. falciparum* 3D7 (chloroquine-sensitive strain) and Dd2 (multidrug-resistant, derived from W2 strain), gametocyte-stage of *P. falciparum* NF54 strain was experimentally at single point concentration of 5 μM, and 10 μM for and ookinetes-stage *P. berghei* and liver schizonts stages (Figure 5). In all stages tested, the compound LDT-695 inhibited ≥50% of parasite growth. Compounds LDT-694, LDT-696, LDT-701, LDT-706, and LDT-708, inhibited ≥50% of parasite growth in at least two stages. According to this analysis, at least six promising compounds from our virtual screening have multistage activity: compounds LDT-694, LDT-695, LDT-696, LDT-701, LDT-706, and LDT-708.

**Figure 5:**
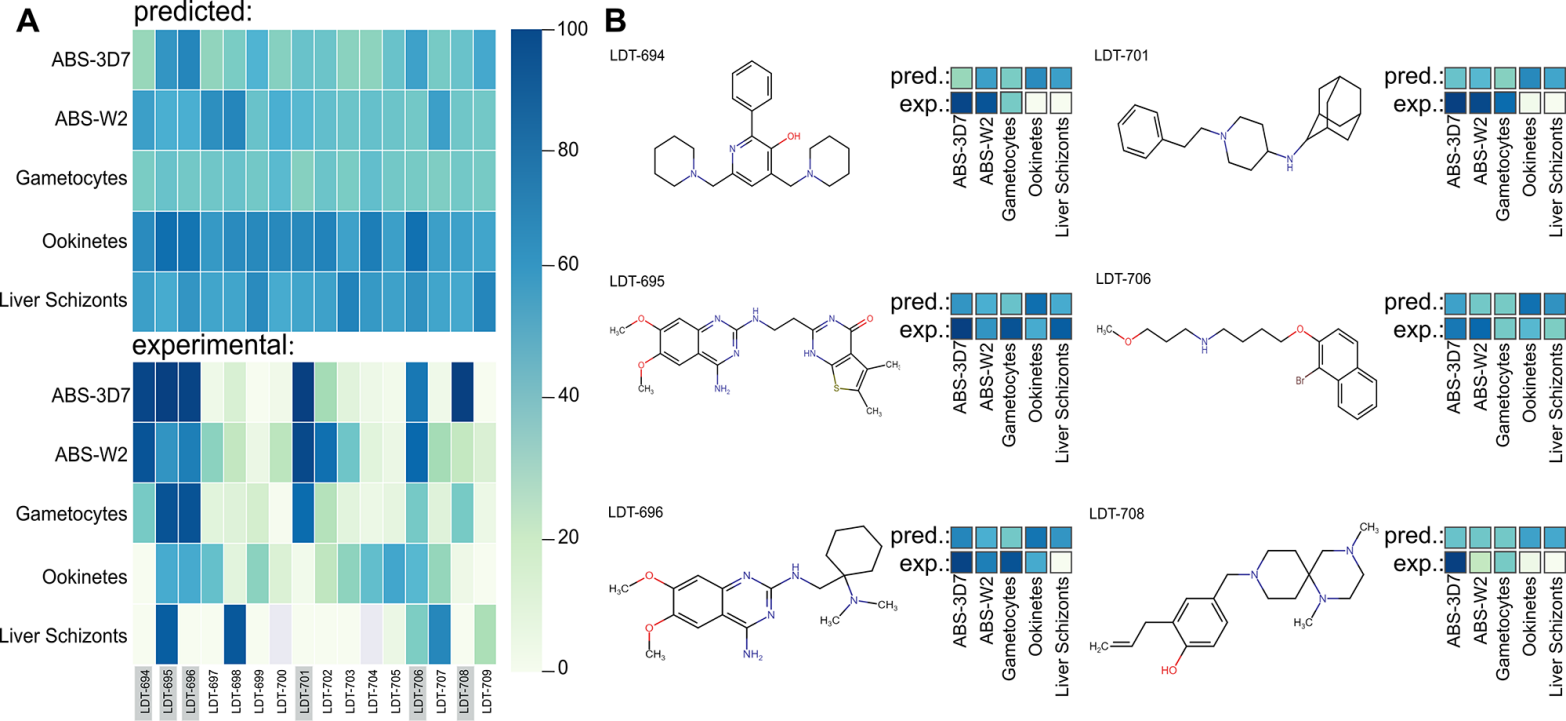
A) Heat maps illustrating the predicted probability of activity (upper heat map) and experimental biological activity profiles (lower heat map) of the selected 16 virtual hits. The probability of activity, predicted by ML-QSAR models, ranges from 0% (light green) to 100% (dark blue). Phenotypic experimental screening was conducted using single-point concentrations against various stages of *Plasmodium spp.*, including asexual blood-stage 3D7 and Dd2 strains, gametocytes, ookinetes, and liver schizonts stages. In these heat maps, dark blue indicates 100% inhibition, while light green represents 0% inhibition. B) The most promising experimental hits with their predicted and experimental profiles. These hits show experimental activity equal to or greater than 50% in at least two *Plasmodium* life stages.

Compounds LDT-695 and LDT-696 showed experimental activity equal to or greater than 50% in five and four models, respectively. This result was anticipated due to their amino quinazoline scaffold, which is known for its activity against blood-stage *P. falciparum* and has also been reported to function as a transmission blocker.^24,25^ This demonstrates the effectiveness of our models in identifying compounds with multistage activity. Interestingly, LDT-706 introduces a novel scaffold and demonstrates moderate activity across all of the *Plasmodium* life cycle stages, making it a promising candidate for hit optimization. Compounds LDT-694, LDT-701, and LDT-706 exhibited 50% or greater inhibitory activity in both ABS and gametocytes stages, classifying them as dual-stage inhibitors. Among these, compound LDT-701 was the most promising, demonstrating over 90% inhibition of ABS in both resistant (Dd2) and sensitive (3D7) strains, and 86% inhibition of the gametocytes stage. Furthermore, compound LDT-698 inhibited parasite growth in liver schizonts stage by over 90%. Although this compound did not exhibit multistage activity, its efficacy against the hepatic stage makes it a candidate for combination therapy aimed at malaria prevention.

Seven compounds (LDT-694, LDT-695, LDT-696, LDT-701, LDT-702, LDT-706, and LDT-708) demonstrated over 50% inhibition of asexual blood-stage parasite growth in both the 3D7 and Dd2 strains at 5 μM. These compounds were further evaluated to determine their dose–response curves and EC_50_ values for both strains. Most compounds exhibited EC_50_ values around 1 μM in the 3D7 strain (Table 2). Notably, LDT-696 and LDT-701 were able to inhibit both the chloroquine-sensitive 3D7 and multidrug-resistant Dd2 strains at a nanomolar scale (Table 2). Additionally, cytotoxicity testing revealed that these compounds were not toxic to HepG2 and COS7 cells, showing favorable selectivity index values.

**Table 2:** Antiplasmodial Activity (EC_50_ and % Inhibition) in Different Strains and Life Cycle Stages of *Plasmodium*, along with Cytotoxicity in Two Mammalian Line Cells (CC_50_), and the Selectivity Index of Selected Compounds^a^

	EC_50_ (μM)		CC_50_ (μM)				% Inhibition		
Compound	*Pf*3D7	*Pf*Dd2	COS7	HepG2	*IS	**IS	Gametocytes Late Stage (IV–V)	Ookinetes	Liver schizonts
**LDT-694**	1.55 ± 0.12	1.65 ± 0.42	>100	>100	>64	>64	51	0	0
**LDT-695**	1.38 ± 0.06	3.67 ± 0.05	12.34 ± 1.2	13.61 ± 3	9	9.9	94	65.02	90.31
**LDT-696**	0.65 ± 0.017	2.65 ± 0.98	51.02 ± 16.6	93.1 ± 2.7	78	142	94	65.29	0
**LDT-697**	11.78 ± 2.95	NT	77.28 ± 23.8	53.1 ± 12	7	4.5	12	56.21	0
**LDT-698**	13.46 ± 8.5	NT	124.63 ± 62.7	54.4 ± 12	9	4	14	11	92.24
**LDT-699**	>10	NT	>100	>100	-	-	20	56	0
**LDT-700**	>10	NT	42.82 ± 7.51	33.24 ± 8.8	-	-	1	45.6	-
**LDT-701**	1.78 ± 0.10	0.43 ± 0.04	48.34 ± 11.1	38.11 ± 4.1	27	21	86	15.97	0
**LDT-702**	5.46 ± 2.1	2.64 ± 1.04	>100	>100	>18	>18	33	3.26	0
**LDT-703**	>10	NT	>100	>100	-	-	11.5	29.12	0
**LDT-704**	>10	NT	51.47 ± 9.2	34.8 ± 1.9	-	-	6	44.70	-
**LDT-705**	>10	NT	56.37 ± 4.2	30.5 ± 1.2	-	-	9.5	56.77	6.96
**LDT-706**	3.04 ± 1.2	1.12 ± 0.66	10.99 ± 0.2	10.4 ± 0.4	2.7	3.4	51.33	66.12	49.36
**LDT-707**	>10	NT	50.42 ± 10.3	44.1 ± 7.3	-	-	13.5	42.67	73.94
**LDT-708**	1.49 ± 0.005	2.59 ± 0.69	>100	>100	>67	>67	51	5.43	0
**LDT-709**	>10	NT	>100	>100	-	-	6	0	0
**ART**	0.002 ± 0.0001	0.0022 ± 0.0005	NT	NT	-	-	NT	NT	NT
**CQ**	0.008 ± 0.002	0.055 ± 0.006	NT	NT	-	-	NT	NT	NT
**PYR**	0.039 ± 0.005	6.01 ± 2.62	NT	NT	-	-	NT	NT	NT

a EC_50_ 3D7 and EC_50_ Dd2: half-maximal effective concentration on asexual blood-stages of *P. falciparum* sensitive and multidrug-resistant strains. CC_50_ COS7 and HepG2, half-maximal cytotoxic concentration on mammalian cells. **SI**: selectivity index (ratio of EC_50_ for 3D7 to CC_50_ for *COS-7 and **HepG-2).% Inhibition of gametocytes late stage (IV–V) in the luciferase assay, using the *P. falciparum* NF54-Luc strain. The data are expressed as the mean ± SD for three independent assays. CQ: Chloroquine. ART: Artesunate. PYR: Pyrimethamine. NT: Not tested.

LDT-695 is a promising multistage compound, exhibiting blood stage EC_50_ values close to 1 μM and over 65% inhibition in the late-stage gametocytes (IV–V), ookinetes, and liver schizonts stages. To further investigate its potential, we utilized SHAP values^20,21^ to assess feature importance, underscoring the compound’s significant promise in antimalarial therapy. Figure 6 depicts the key features of compound LDT-695 identified by each model in the context of “active” prediction. The aminoquinazoline group prominently appeared in the predictions across all stages. This is consistent with previous reports that compounds with this core exhibit activities against the asexual blood stage (in both *P. falciparum* and *P. vivax*), sexual-stage gametocytes, ookinete formation, and liver-stage schizonts.^25−27^ However, noteworthy variations were observed in the specific fragments within this group, contributing to predictions across different models. This variability highlights distinct learning patterns among the models, even when analyzing common structural motifs like aminoquinazoline.

**Figure 6:**
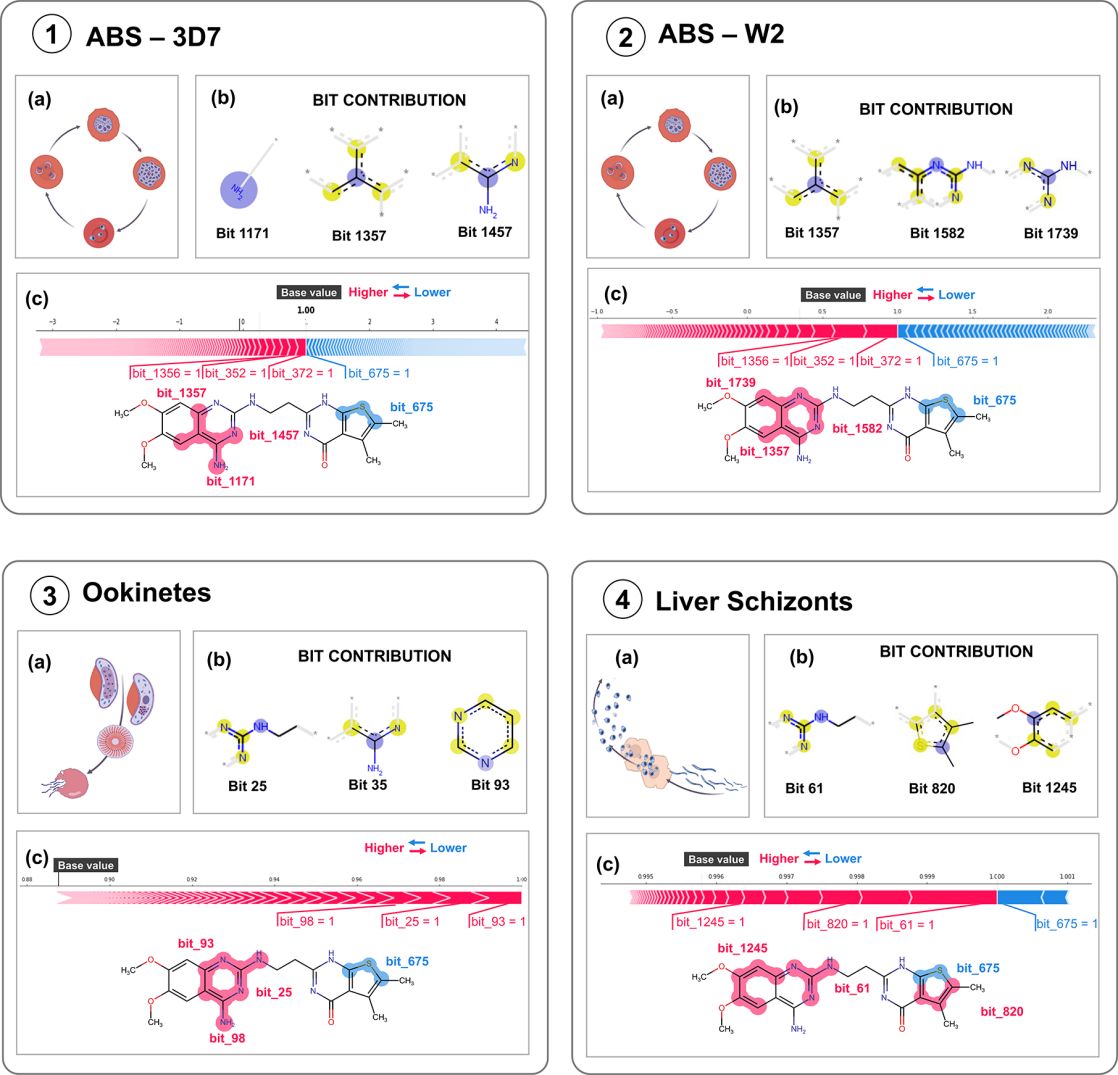
Local SHAP interpretation for the ML-QSAR models on a) each lifecycle stage of plasmodium (1- ABS-3D7, 2- ABS-W2, 3- ookinetes stage, 4- liver schizonts stage) for the compound LDT-695. In the plot, red denotes a positive impact, while blue signifies a negative impact on the model prediction. b) The most important bits on compound LDT-695 contribute to each model’s predictions. Blue contour atoms: represent the central atom in the environment; yellow: aromatic atoms; gray: aliphatic ring atoms. c) Force plots for local SHAP contributions and highlighted fragments in LDT-695 corresponding to the most frequent bits.

An intriguing finding was the consistent prediction of the potential impact of sulfur in the thiophene group on the antimalarial activity across all models. In the liver schizont stage model, for instance, the thiophene group (illustrated by bit 820) was noted for its positive impact on activity. Notably, the bit centroid represents a carbon atom adjacent to the sulfur atom, while the sulfur component itself (depicted by bit 675) exerted a negative influence within the same model. This dual influence underscores the complex interaction between molecular components, suggesting a potential for optimizing sulfur atom substitution. Overall, this analysis reveals promising opportunities for refining compound design to enhance therapeutic efficacy driven by insights into molecular interactions gleaned from model predictions.

In conclusion, this study addresses the urgent global health challenge posed by malaria through the introduction of a comprehensive suite of multistage ML-QSAR models. These models effectively assess the activity of chemical compounds against distinct life stages of *Plasmodium* parasites. Our research not only led to the development of robust QSAR models but also involved a rigorous external validation process, affirming their reliability and utility. Furthermore, we identified promising compounds with broad-spectrum inhibitory potential across all five malaria life stages. The elucidation of crucial molecular fragments pivotal to model predictions enhances our understanding of these models’ mechanisms, making them more transparent and interpretable for future research endeavors. Ultimately, these models have the potential to expedite the discovery and optimization of multistage compounds, providing valuable tools to advance malaria elimination strategies globally. In an era where innovative antimalarial therapies are urgently needed to combat rising drug resistance, this research contributes significantly to ongoing efforts aimed at alleviating the burden of this devastating disease.

## Data Availability

All scripts and databases utilized in this study are available at https://github.com/LabMolUFG/malaria_multistage.

## Supporting Information Available

Detailed computational procedures, experimental procedures, supplementary figures, and characterization of compounds (PDF)
