# Supplementary material for: Innovative Multistage ML-QSAR Models for Malaria: From Data to Discovery
Source: ACS Med Chem Lett. 2024 Jul 18;15(8):1386–95. doi: 10.1021/acsmedchemlett.4c00323 (PMC11318017; doi:10.1021/acsmedchemlett.4c00323)
Supplement: Supplementary file 1 — ml4c00323_si_001.pdf [file ml4c00323_si_001.pdf]

Innovative Multi-stage ML-QSAR Models for Malaria: From Data to Discovery

Joyce V. B. Borba[1-4], Luis Carlos Salazar[1], Leticia Tiburcio1, Sabrina Silva-Mendonça[2-4], Meryck Felipe Brito da Silva[2-4], Igor H. Sanches[2-4], Aline Rimoldi[1], Juliana Calit[5], Ana Sofia Pinto Santana[7], Miguel Prudêncio[7], Pedro V. Cravo[8], Daniel Y. Bargieri[5], Gustavo C. Cassiano[8], Fabio T. M. Costa[1*] Carolina H. Andrade[2-4*]

[1] Laboratory of Tropical Diseases – Prof. Dr. Luiz Jacintho da Silva, Department of Genetics Evolution, Microbiology and Immunology. Institute of Biology, UNICAMP, 13083-970 Campinas, SP, Brazil.

[2] Laboratory for Molecular Modeling and Drug Design (LabMol), Faculty of Pharmacy, Federal University of Goias, Rua 240, qd. 87, Goiânia, GO 74605-170, Brazil.

[3] Center for Excellence in Artificial Intelligence (CEIA), Institute of Informatics, Universidade Federal de Goiás, Goiânia, 74605-170, GO, Brazil.

4 Center for the Research and Advancement in Fragments and Molecular Targets (CRAFT), School of Pharmaceutical Sciences at Ribeirao Preto, University of São Paulo, Ribeirão Preto, SP, Brazil.

5 Department of Parasitology, Institute of Biomedical Sciences, University of São Paulo, 05508-000, São Paulo, SP, Brazil.

7 Instituto de Medicina Molecular Jão Lobo Antunes, Faculdade de Medicina da Universidade de Lisboa, Lisboa, Portugal

8 Global Health and Tropical Medicine, Associate Laboratory in Translation and Innovation Towards Global Health, Instituto de Higiene e Medicina Tropical, Universidade NOVA de Lisboa, Rua da Junqueira 100, 1349-008 Lisbon, Portugal

* Corresponding authors: carolina@ufg.br; fabiotmc72@gmail.com

Table of Contents

Computational procedures S3
Experimental Procedures S7
Supplementary Figures S10
Compound characterization...S11
References S23

Computational procedures

- **Data collection and curation**

Datasets were thoroughly curated following previously developed workflows (Fourches et al. 2016). First, we excluded inconsistent data such as experimental results without EC_{50} determination or with a not precise EC_{50} value ("bigger then" - >, "smaller then" - <), HTS data with not trustable and low efficiency curves. Then we removed mixtures, inorganics, and organometallic compounds, cleaned and neutralized salts, normalized the specific chemotypes, and performed a duplicate analysis as follows: (i) when replicated records presented the same binary outcome, only one record was kept; (ii) when the majority of replicate chemicals presented the same binary outcome and one had different binary outcome, only one record with the most common binary outcome was kept, (iii) when replicated records had different binary outcomes, all of them were removed. All the curated data are available in the Supplemental Material.

- ***ABS-3D7 dataset***

The data from blood stage sensitive strain 3D7 (ABS-3D7) were compiled from the publicly available databases PubChem[1] (IDS: AID660866, AID524790, AID1828, AID652047, AID504832, AID449703) and ChEMBL[2] (IDS: CHEMBL1267245, CHEMBL1837547, CHEMBL2028071, CHEMBL2366922, Chembl_NTD Pathogen_box, Chembl_NTD Medicine for Malaria Venture, and Chembl_NTD Saint Jude); and from the literature[3]. A total of 14 datasets were compiled with a total of 129,411 records of compounds tested against 3D7 strain. After removing inconsistent data 42,502 records remained. After removal of mixtures, inorganics, and neutralization of counter-ions, 42,405 records remained. All the discordant replicates and one of each concordant replicates were removed. The final dataset has 35,943 unique chemical compounds, including 15,208 active and 20,735 inactive compounds. The threshold used for classifying compounds as active was $EC_{50} < 1$ μM.

- ***ABS-W2 dataset***

The data from blood stage resistant strain W2 (ABS-W2) were compiled from the publicly available databases PubChem[1] (IDS: AID1883, AID449704, AID449720, AID606570, AID616029, AID524796) and ChEMBL[2] (IDS: CHEMBL1261024, CHEMBL1261025, CHEMBL1687072, CHEMBL3369904). A total of 10 datasets were compiled with a total of 7,982 records of compounds tested against W2 strain. After removing inconsistent data 6,552 records remained. After removal of mixtures, inorganics, and neutralization of counter-ions, 6,331 records remained. At this point in the curation pipeline, the dataset had 3,362 active

compounds and 2,968 inactive compounds. We decided to add 968 inactive compounds from the 3D7 dataset to balance the data. All the discordant replicates and one of each concordant replicates were removed. The final dataset has 6,724 unique chemical compounds, including 3,362 active and 3,362 inactive compounds. The threshold used for classifying compounds as active was $EC_{50} < 1$ µM.

- ***Gametocyte dataset***

The data from gametocyte assays were compiled from the publicly available databases PubChem[1] (ID: AID1117278) and ChEMBL[2] (IDS: CHEMBL3800921, CHEMBL3832784, CHEMBL3996812, CHEMBL2439657, CHEMBL3297427, CHEMBL3389350, CHEMBL4231440, CHEMBL4268539, CHEMBL4268540, CHEMBL3301555, CHEMBL3802513, CHEMBL3996814); and from the literature[4,5]. A total of 17 datasets were compiled with a total of 739 records of compounds tested against gametocytes. We only selected data on late-stage gametocytes (stage V). After removing inconsistent data 696 records remained. After removal of mixtures, inorganics, and neutralization of counter-ions, 695 records remained. All the discordant replicates and one of each concordant replicates were removed. The final dataset has 558 unique chemical compounds, including 166 active and 392 inactive compounds. The threshold used for classifying compounds as active was $EC_{50} < 1$ µM.

- ***Ookinete dataset***

Our collaborator generously provided the data from ookinete stage assays, involving the testing of 6,636 compounds. Only those exhibiting inhibition below 2 µM (344 compounds) underwent re-testing in a confirmatory assay. Both the initial and confirmatory sets were subjected to the same curation process, involving the removal of inconsistent data (efficacy below 70% and positive curve class). This resulted in curated sets of 1,496 and 332 records, respectively. Further refinement included the removal of mixtures, inorganics, and neutralization of counter-ions, leaving 1,493 and 328 records. Subsequently, inactive compounds from the first curated set were added to the confirmatory set. Analyzing the united data involved addressing discordant replicates by analyzing and removing duplicates, while retaining only one of each concordant replicate. The final dataset comprises 1,562 unique chemical compounds, consisting of 293 active and 1,269 inactive compounds. The classification criterion for active compounds was an EC50 below 2 µM, following Katsuno et al.

- ***Liver dataset***

The data from liver-stage assays were compiled from the publicly available database ChEMBL[2] (IDS: CHEMBL2092592, Chembl_NTD Pathogen_box, Chembl_NTD

UCSD_MMV_Box, Chembl_NTD Novartis), and from the literature[6]. A total of 6 datasets were compiled with a total of 1,344 records of compounds tested against liver-stage *Plasmodium*. After removing inconsistent data (no defined EC50, only one tested concentration, wrong measurement units) 1,221 records remained. After removal of mixtures, inorganics, and neutralization of counter-ions, 1,218 records remained. All the discordant replicates and one of each concordant replicates were removed. The final dataset has 1,189 unique chemical compounds, including 523 active and 666 inactive compounds. The threshold used for classifying compounds as active was $EC_{50} < 1$ µM.

- **Chemical Space Analysis**

For each dataset, Murcko scaffolds were calculated, compounds with the same scaffold were grouped, and their activity in each group was averaged. A *t*-distributed stochastic neighbor embedding (*t*-SNE)[7] analysis using ECFP4[8] descriptors with 2024 bits available on RDkit[9] package v. 2022.03.2 was performed in Python v3.10 to visualize the chemical space of the data set's scaffolds.

- **Machine Learning-based QSAR**

Binary QSAR models were developed and rigorously validated according to the best practices of QSAR modeling[10]. Two-dimensional ECFP fingerprints[8], FCFP fingerprints[11] and MACCS keys[12], calculated with RDKit package[9], and Mordred, calculated with Mordred package for Python[13], were combined with Random Forest[14] algorithm implemented in scikit-learn[15] for model building. The models were optimized using a Bayesian approach implemented in Scikit-Optimize v.0.7.4.[16]. The ML-QSAR models were calibrated using a threshold-moving approach implemented in Scikit-learn v.0.24.2.[15] This approach uses different probability thresholds in the range of 0 to 1 obtained via the Receiver Operating Characteristic (ROC) curve to find the threshold with the largest G-mean value.

Datasets were split into modeling sets (80% of compounds) and external sets (20% of compounds) using the random distribution approach. The modeling sets were used to generate QSAR models through a 5-fold cross-validation (5FCV) approach where statistics for each endpoint were calculated by averaging the statistics of the models for each fold (Alves et al. 2015), while the external sets were used to assess the predictive power of models. The AD (Applicability Domain) model was developed considering only predictions from individual models when the queried compound was inside the applicability domain, using the *z*-cutoff method[17]. In every case, only the modeling set was used to develop the models, while the external sets were used for the evaluation of their predictive power. In addition, ten rounds of Y-randomization were performed for each dataset to assure that the models' performance was

not due to chance correlations. The Shapley Additive Explanations (SHAP) [18,19] technique was employed to understand model predictions.

The predictive performance of QSAR models was evaluated using balanced accuracy (BACC), sensitivity (SE), specificity (SP), precision, F1 score, and Mattew's correlation coefficient (MCC).(equations 1-7):

$$SE = \frac{TP}{TP + FN} \quad [Eq. 1]$$

$$SP = \frac{TN}{TN + FP} \quad [Eq. 2]$$

$$BACC = \frac{SE + SP}{2} \quad [Eq. 3]$$

$$Precision = \frac{TP}{TP + FP} \quad [Eq. 4]$$

$$Recall = \frac{TP}{TP + FN} \quad [Eq. 5]$$

$$F1 = \frac{2x \; precision \; x \; recall}{precision + recall} \quad [Eq. 6]$$

$$MCC = \frac{TNxTP - FPXFN}{SQRT((TN + FN)(FP + TP)(TN + FP(FN + TP))} \quad [Eq. 7]$$

where N represents the number of compounds, TP and TN represent the number of true positives and true negatives, and FP and FN represent the number of false positives and false negatives, respectively.

- **Virtual Screening and selection of new compounds**

Developed multi-stage QSAR-ML models were used for VS of the commercially available ChemBridge database (http://www.chembridge.com/) v. 2020, aiming to identify new potential multiple stage antiplasmodial compounds. The database was filtered using the five QSAR-ML models in the following order: ABS-3D7, ABS-W2, ookinete, gametocyte, and liver stage. Then, drug likeness of the remaining compounds was evaluated using Veber[20] and Lipinski's rules[21]. In addition, the structural diversity of candidate compounds was investigated using a cluster analysis where compounds were grouped into clusters of similar compounds and selected by visual inspection. We also conducted a literature search to check if the compounds have been previously tested against *Plasmodium* sp. Parasites. Finally, the selected candidate compounds were purchased and submitted to in vitro experimental evaluation.

Experimental procedures

- ***Plasmodium falciparum* culture and antiplasmodial activity.**

The *in vitro* antiplasmodial activity assay was assessed on *P. falciparum* 3D7 (chloroquine sensitive) and Dd2 (chloroquine resistant) strains (Wellems and Plowe 2001). The parasites were cultivated in RPMI 1640 medium (SIGMA-ALDRICH), supplemented with hypoxanthine 0.005%, glucose 0.2%, sodium bicarbonate 0.2%, O^+ red blood cells (RBCs) and A^+ human plasma 10%. The cultures were incubated at 37°C in a low oxygen environment (3% O2, 5% CO2, and 92% N2) as previously described by Trager et al.[22]. Drug inhibition assays were performed as previously described[23]. Briefly, synchronizations with 5% D-Sorbitol solution were performed at 48 h intervals before the experiments, to allow incubations with >90% of the parasites in the ring stage. Assays were performed in a 96-well plate, with 0.5% parasitemia and 2% hematocrit, in the presence of 5 µM of compounds or the drug vehicle (DMSO), as a control. For determination of 50% effective concentrations (EC_{50}), parasites were incubated in the presence of drugs in a two-fold 12-point serial dilution starting at 10 µM in duplicate. Chloroquine was used as an antimalarial standard. After 72 h of incubation, parasitemia was assessed by fluorometry using SybrGreen fluorescent dye. The plates were read in a CLARIOstar plate reader (BGMtech) by fluorescence at 490 nm excitation and 540 nm emission wavelengths. The growth inhibition values were expressed as percentages relative to the drug-free control. EC_{50} values were interpolated from log doses vs. inhibition curves in GraphPad Prism 8. The experiments were carried out in three independent assays.

- **Toxicity evaluation in mammalian cell lines**

The compound's cytotoxicity evaluation was performed in two mammalian cells lines: monkey kidney tissue-derived fibroblast cell line (COS-7) and human hepatoma cell line (HepG2). The 3-[4,5-dimethyl-thiazol-2-yl]-2,5-diphenyltetrazolium bromide (MTT) assay was performed as previously described (Kumar et al. 2018). Briefly, COS-7 and HepG2 cells were maintained in DMEM (SIGMA-ALDRICH) at 37 °C in a humidified atmosphere containing 5% CO_2. The culture media was supplemented with 10% fetal bovine serum (FBS) and 1% L-glutamine, penicillin-streptomycin solution. The cells were plated in 96-well plates at a concentration of 10^4 cells per well and incubated for 16h to get 70% of confluence, then the cells were incubated for 72 h with serial dilutions of compounds (100 to 0.00038 µM). Absorbance was accessed at an optical density of 570 nm (OD_{570}) in a CLARIOstar plate reader (BGMtech), and the percent viability of cells was expressed as a percentage relative to the untreated control.

- **Gametocytocidal activity assay**

Gametocytes were obtained from the *P. falciparum* NF54-Pfs16-GFP-Luc (MRA-1217) (named here NF54-Luc) strain[24]. NF54-Luc asexual parasites were cultivated as described in section 1.1. Synchronizations were performed with a 5% D-Sorbitol solution to obtain ring-stage parasites. Then, the parasites were cultured until reached 8–10% parasitemia at rings-stages, when the process of gametogenesis induction through two steps of nutrient deficit stress was started, following the protocol described by Fivelman *et al*[25], with some modifications. Briefly, when the culture reached the desired parasitemia in rings-stages, only 40% of the medium was renewed causing the first stress, followed by a second stress (keeping 40% of the old media on the consecutive day) in trophozoite stages. After stress induction, the culture was maintained for 5 days with a complete medium containing 50 mM of N-acetyl glucosamine (GlcNAc) (SIGMA-ALDRICH) to inhibit merozoite invasion and thus have a culture without contamination by asexual stages. Then, the medium was changed daily (without GlcNAc) until obtaining mature gametocytes. For the isolation of mature gametocytes (stage IV-V) among residual RBCs, we used the MACS® cell separation column (Miltenyi Biotech, Germany). Assays were performed in 96-well plates, two independent experiments in duplicate, containing $2x10^5$ gametocytes per well in the presence of 5 µM of compounds or Methylene Blue (MB) as control. The plate was incubated in a gas chamber for 48 hours at 37°C under the same culture conditions. After incubation, the luciferase activity was determined in 20 µL of parasite lysates, adding 50 µL of luciferin substrate (Promega Luciferase Assay System) at room temperature according to the manufacturer's recommendations. The bioluminescence was read at an integration constant of 10s with BioTek Cytation 5 Cell Imaging Multimode Reader with Gen5 Software. The results were expressed as a percentage of gametocyte inhibition compared to the untreated gametocytes using GraphPad Prism 8 software.

- **Ookinete activity assay**

To evaluate the activity of the compounds against the sexual ookinete stage of *P. berghei*, we employed the PbOokluc murine model, as described by Calit et al.[26]. Initially, the compounds were diluted in ookinete medium[27] to a final concentration of 10 µM and a total volume of 40 µL. Then, 4 µL of blood from mice infected with the PbOokluc strain, containing gametocytes, were added, and incubated at 21ºC for 24 hours. After this incubation period, nanoluciferase activity was measured using a plate luminometer.

- **Liver stage assay**

The assessment of compound activity in the liver stage of *P. berghei* expressing firefly luciferase involved the quantification of luminescence, as detailed in a previous study[28].

Briefly, 10^4 sporozoites obtained through dissection of infected *Anopheles stephensi* were added into individual wells of a 96-well plate with Huh-7 cells (10^4 cells per well). Compounds were added one hour prior to infection. After 46 hours of exoerythrocytic growth at 37°C and 5% CO_2, the parasite load was quantified using a bioluminescence assay (Biotium). The viability of Huh-7 cells exposed to the compounds was evaluated using the AlamarBlue assay (Invitrogen) before measuring bioluminescence. DMSO and atovaquone served as negative and positive controls, respectively. The percentage of infection was determined relative to the control, and EC_{50} values were calculated using Prism GraphPad software.

Supplementary Figures

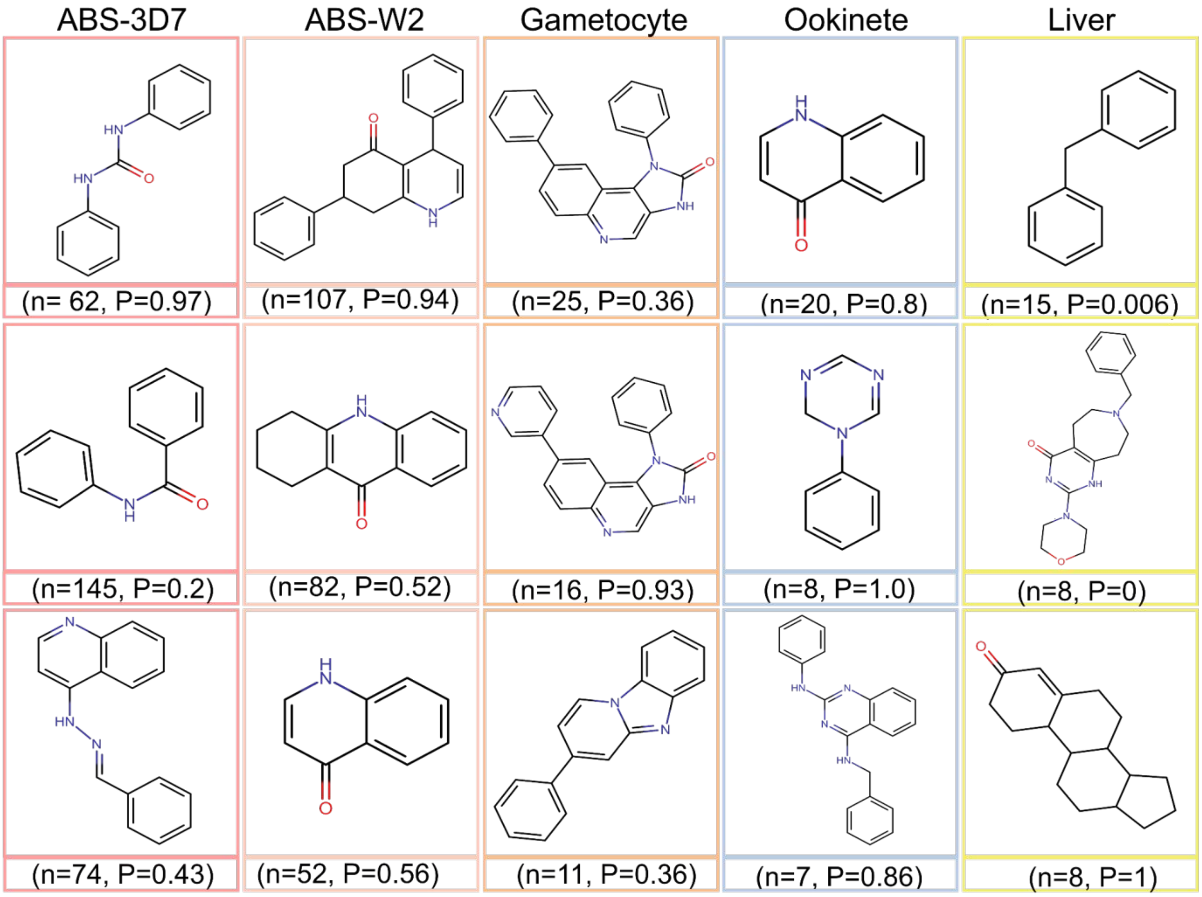

Figure S1: Most prevalent scaffolds within each dataset. 'n' represents the total number of compounds featuring the scaffold, while 'P' denotes the probability of encountering an active compound with that scaffold, ranging from 0 to 1.

Compound characterization

LDT-694

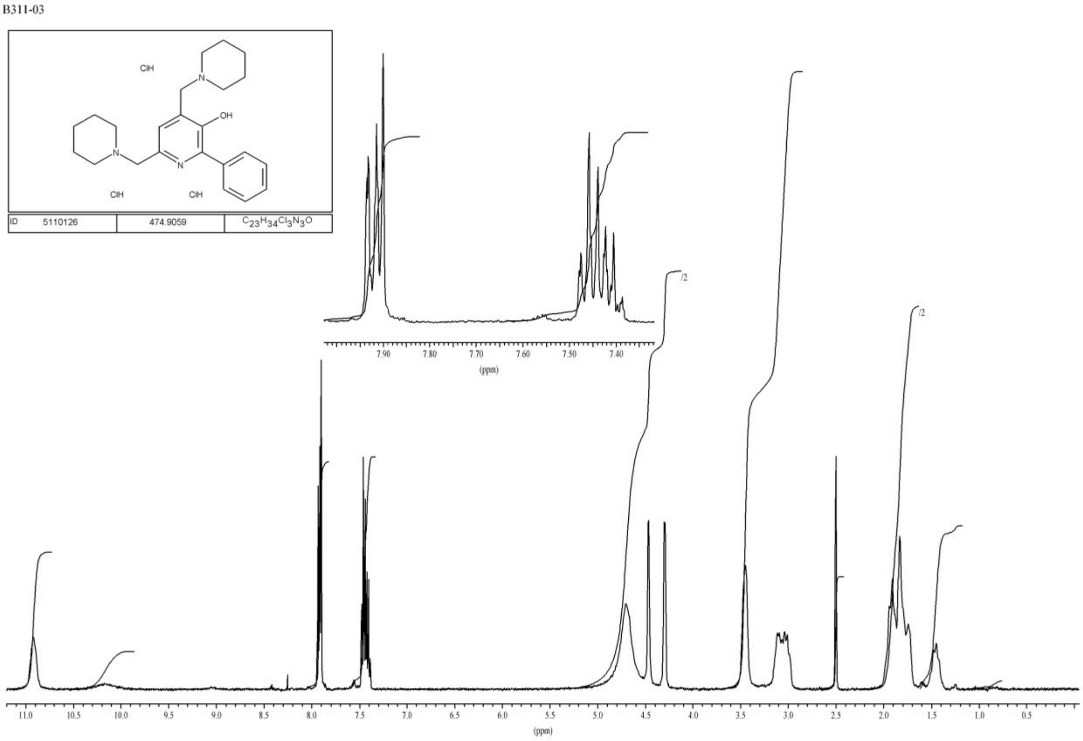

LDT-695

FC942501745

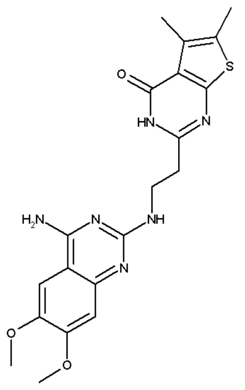

ID	11204988	426.5007	$C_{20}H_{22}N_6O_3S$

```
Data File D:\DATA\51\2EF-4501.D
Sample Name: FC9425017P2-E-06
Instrument 1 06/04/2018 22:59:12  6
Column: Onyx Monolithic C18 50x4.6mm | 3.75ml/min
| Columns Reg Valve
Gradient: "A"->@2.2min->"B"(Hold 0.4min)-
>@0.2min->"A"->PostRun
PMP1, Solvent A          : 0.1%TFA in Acn/H2O
(2.5:97.5)
PMP1, Solvent B          : 0.1%TFA in AcN
PMP1, Solvent C          : 0.1%FA in Acn/H2O
(2.5:97.5)
PMP1, Solvent D          : 0.1%FA in AcN
Ionization mode               :    APCI Positive

Signal 1: ADC1 B, ELSD
Peak RetTime Type  Width     Area      Height
Area
  #   [min]         [min]   [mAu*s]     [mAu]
%
----|-------|----|-------|----------|----------|-
-------|
   1   1.111 BB    0.0218   52.20713   38.66664
100.0000
Totals :                    52.20713   38.66664


Signal 2: DAD1 A, Sig=300,200 Ref=off
Peak RetTime Type  Width     Area      Height
Area
  #   [min]         [min]   [mAU*s]     [mAU]
%
----|-------|----|-------|----------|----------|-
-------|
   1   1.071 BB    0.0280 2151.90039 1253.29065
100.0000
Totals :                  2151.90039 1253.29065


Signal 3: MSD1 TIC, MS File
Peak RetTime Type  Width     Area      Height
Area
  #   [min]         [min]
%
----|-------|----|-------|----------|----------|-
-------|
   1   1.096 PB    0.0441 5.88486e5   2.08768e5
100.0000
Totals :                  5.88487e5   2.08768e5
```

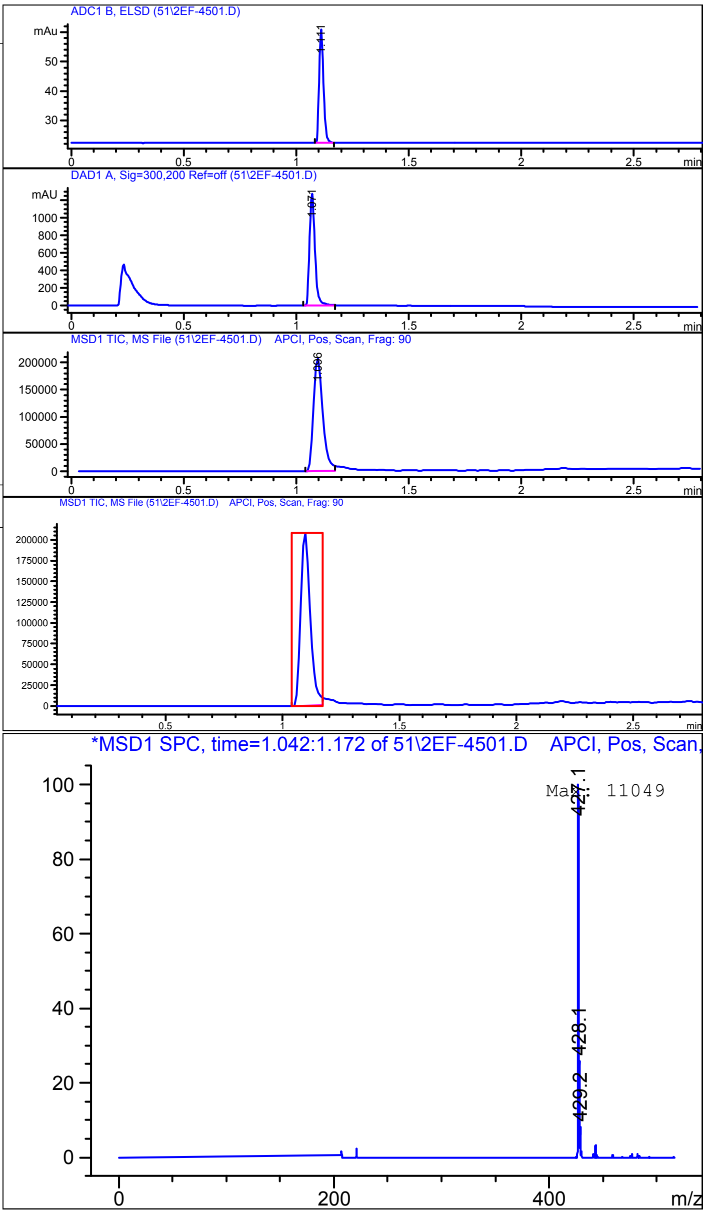

LDT-696

FC942490229

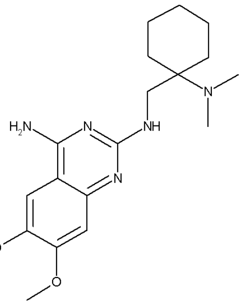

ID	79791450	359.4753	$C_{19}H_{29}N_5O_2$

Data File D:\FC9424~1\1ED-2201.D
Sample Name: FC9424902P1-E-04
Instrument 1 29/03/2018 11:22:21
Column: Onyx C18 50x4.6mm | 3.75ml/min | Columns Reg Valve
Gradient: "A"->@2.0min->"B"(Hold 0.6min)->@0.2min->"A"->PostRun
PMP1, Solvent A : 0.1%TFA in Acn/H2O (2.5:97.5)
PMP1, Solvent B : 0.1%TFA in AcN
PMP1, Solvent C : 0.1%FA in Acn/H2O (2.5:97.5)
PMP1, Solvent D : 0.1%FA in AcN
Ionization mode : APCI Positive

Signal 1: ADC1 B, ELSD

Peak #	RetTime [min]	Type	Width [min]	Area [mAu*s]	Height [mAu]	Area %
1	0.963	BB	0.0263	61.64272	37.21132	100.0000
Totals :				61.64272	37.21132	

Signal 2: DAD1 A, Sig=300,200 Ref=off

Peak #	RetTime [min]	Type	Width [min]	Area [mAU*s]	Height [mAU]	Area %
1	0.918	MM	0.0329	2153.78638	1090.62939	94.5386
2	1.088	MM	0.0389	66.68647	28.60078	2.9271
3	1.250	MM	0.0351	57.73565	27.38428	2.5343
Totals :				2278.20849	1146.61446	

Signal 3: MSD1 TIC, MS File

Peak #	RetTime [min]	Type	Width [min]	Area	Height	Area %
1	0.944	BB	0.0468	1.26744e6	4.50812e5	100.0000
Totals :				1.26744e6	4.50812e5	

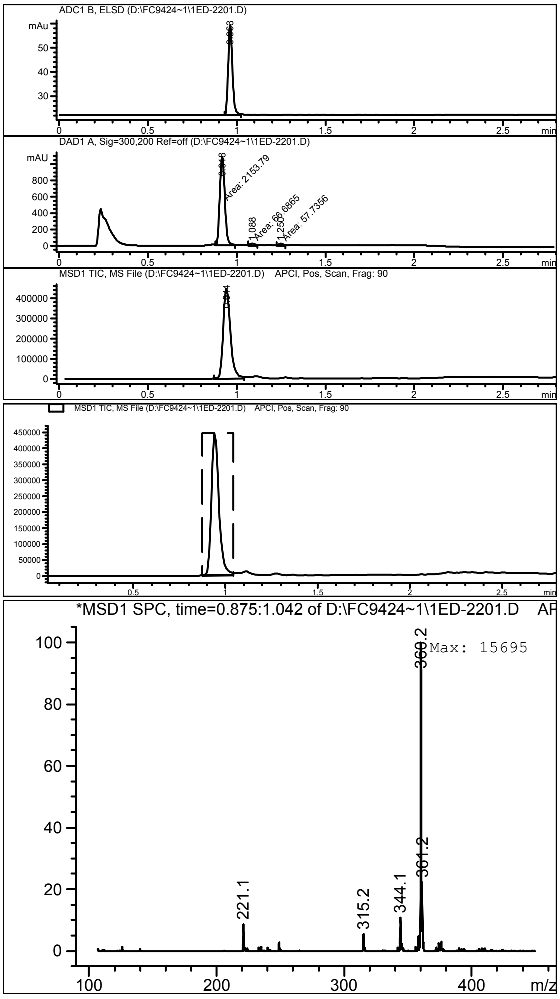

LDT-697

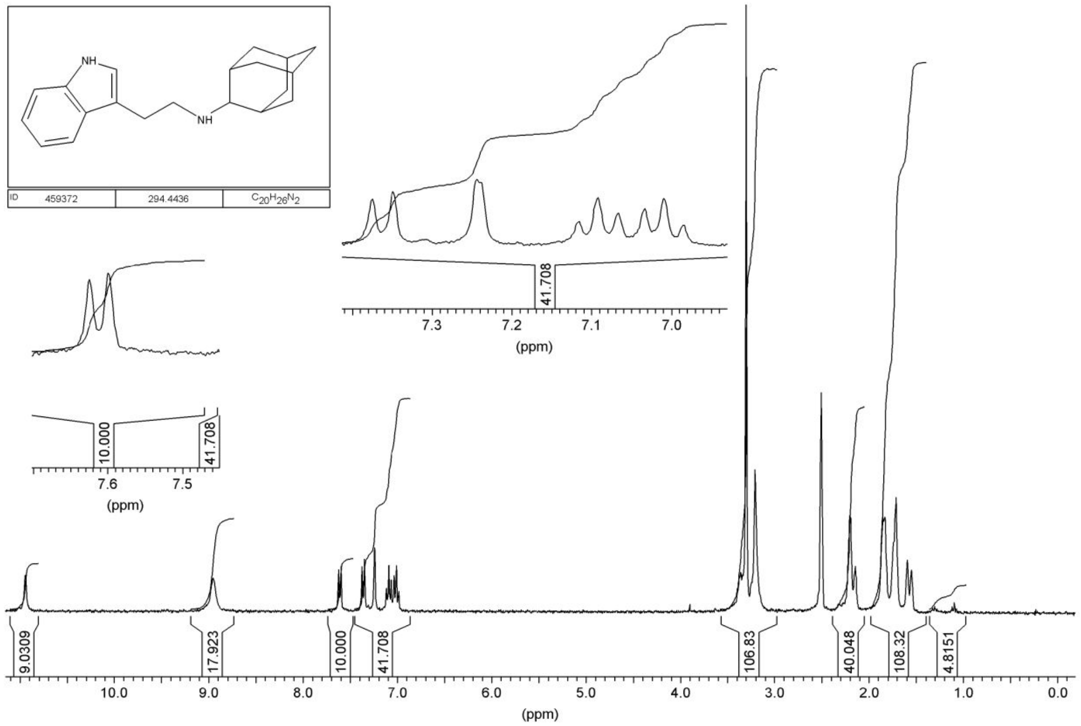

LDT-698

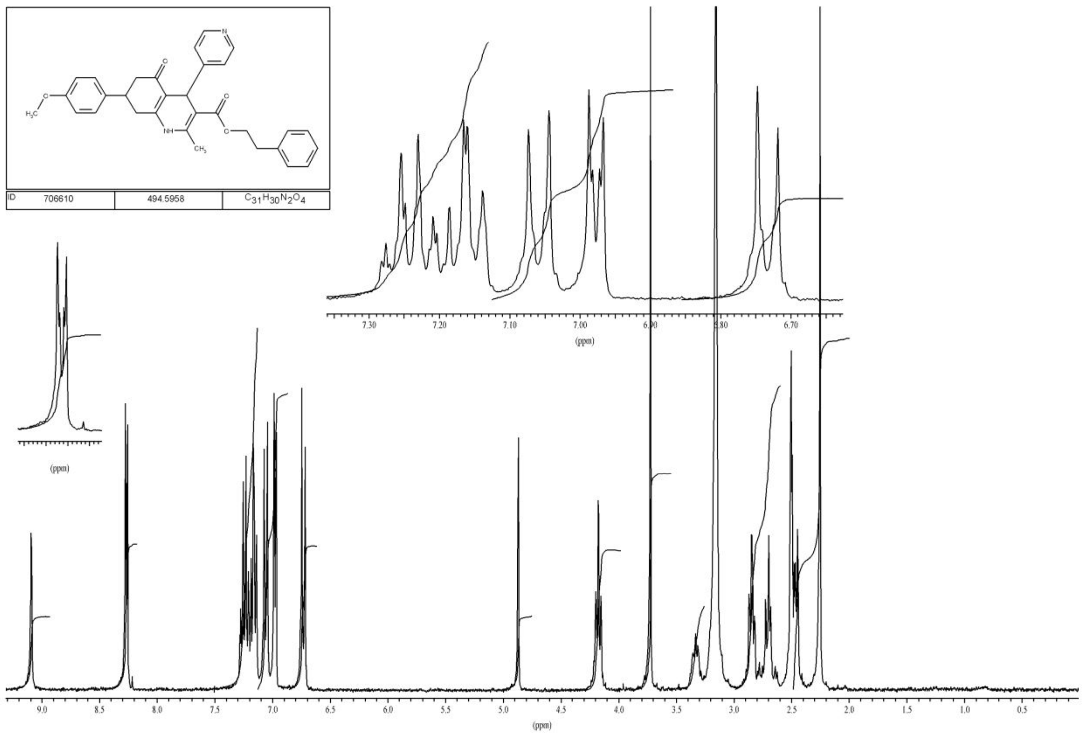

LDT-699

ST1147605		
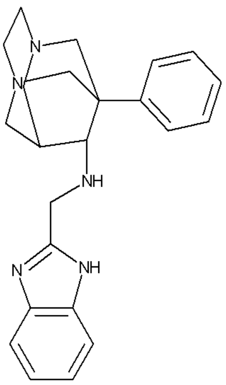		
ID 76794652	373.5051	$C_{23}H_{27}N_5$

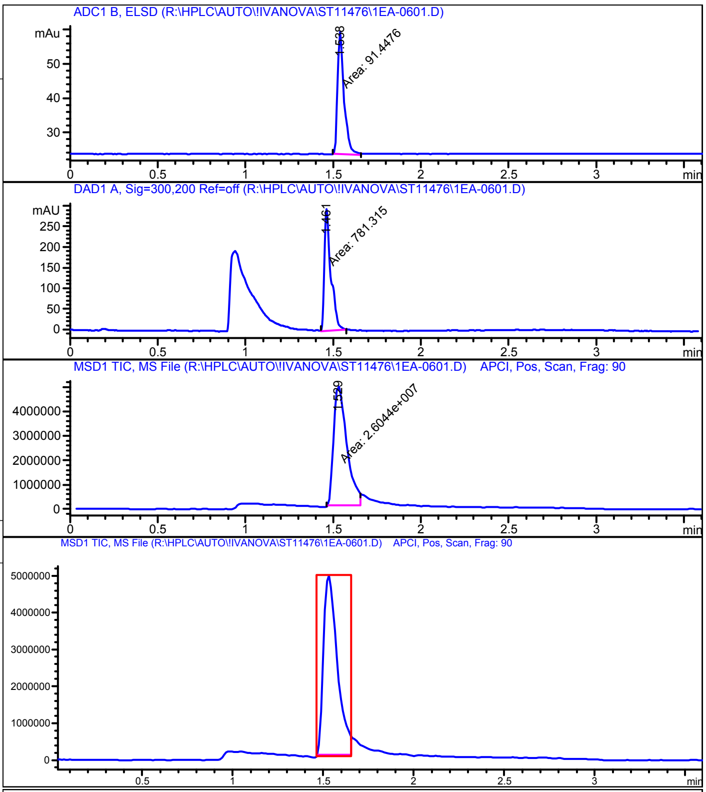

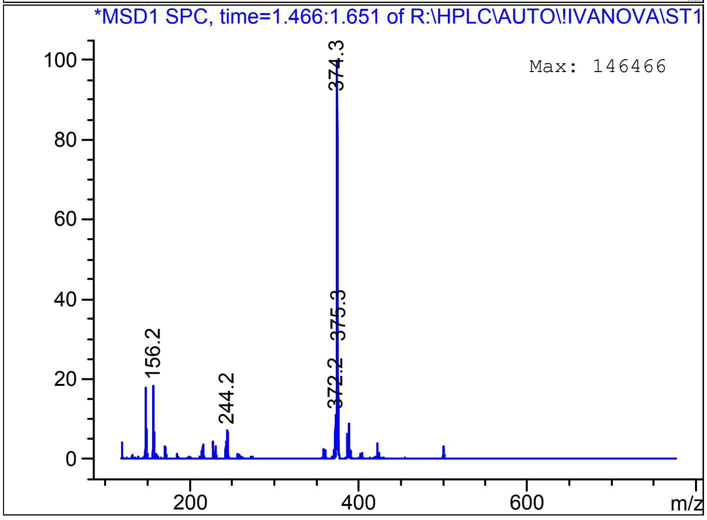


```
Data File R:\HPLC\AUTO\!IVANOVA\ST11476\1EA-
0601.D
Sample Name: ST11476P1-E-01
Instrument 1 01/09/2022 16:46:12 PM
Injection Date  : 07/08/2022 3:08:45
Column:  C18 50x2.1mm | 0.80ml/min | Columns Reg
Valve
Gradient: "A"->@2.2min->"B"(Hold 0.4min)-
>@0.01min->"A"(Hold 1.0min)->PostRun
PMP1, Solvent A          : 0.1%TFA in Acn/H2O
(2.5:97.5)
PMP1, Solvent B          : 0.1%TFA in AcN
PMP1, Solvent C          : 0.1%FA in Acn/H2O
(2.5:97.5)
PMP1, Solvent D          : 0.1%FA in AcN
Ionization mode               :    APCI Positive

Signal 1: ADC1 B, ELSD
Peak RetTime Type  Width     Area      Height
Area
  #   [min]        [min]   [mAu*s]     [mAu]
%
----|-------|----|-------|----------|----------|-
-------|
   1   1.538 MM    0.0420   91.44761   36.31004
100.0000
Totals :                    91.44761   36.31004


Signal 2: DAD1 A, Sig=300,200 Ref=off
Peak RetTime Type  Width     Area      Height
Area
  #   [min]        [min]   [mAU*s]     [mAU]
%
----|-------|----|-------|----------|----------|-
-------|
   1   1.461 MM    0.0443  781.31488  294.07092
100.0000
Totals :                   781.31488  294.07092


Signal 3: MSD1 TIC, MS File
Peak RetTime Type  Width     Area      Height
Area
  #   [min]        [min]
%
----|-------|----|-------|----------|----------|-
-------|
   1   1.529 MM    0.0888 2.60440e7  4.88809e6
100.0000
Totals :                  2.60440e7  4.88809e6
```

LDT-700

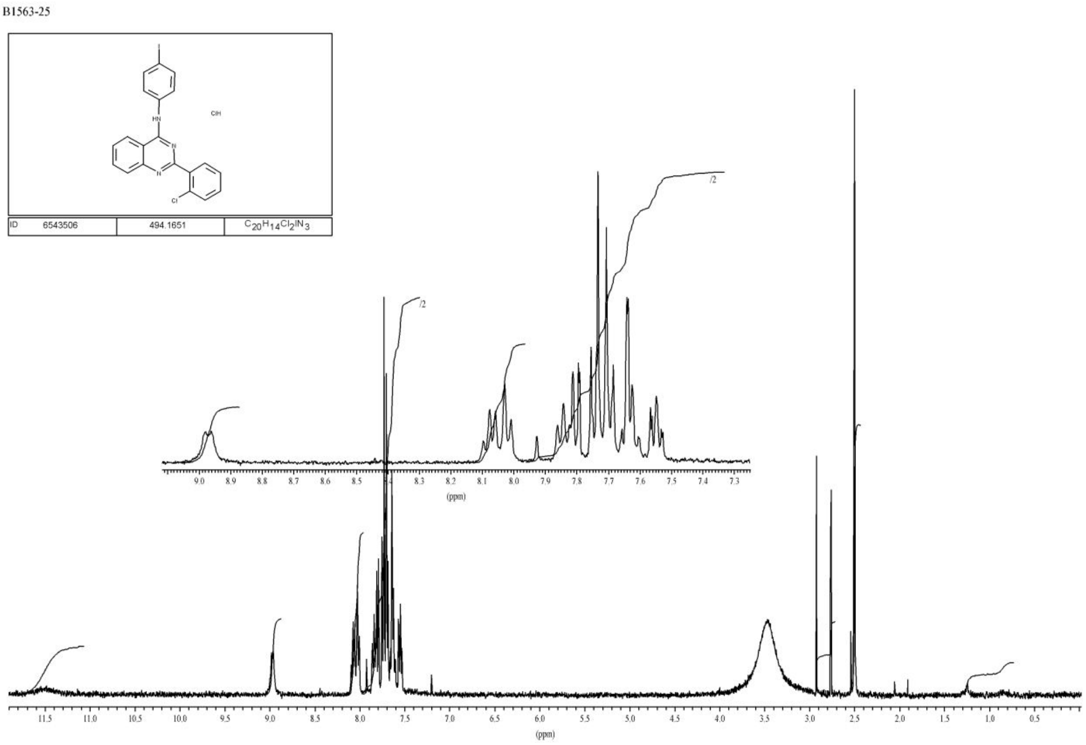

LDT-701

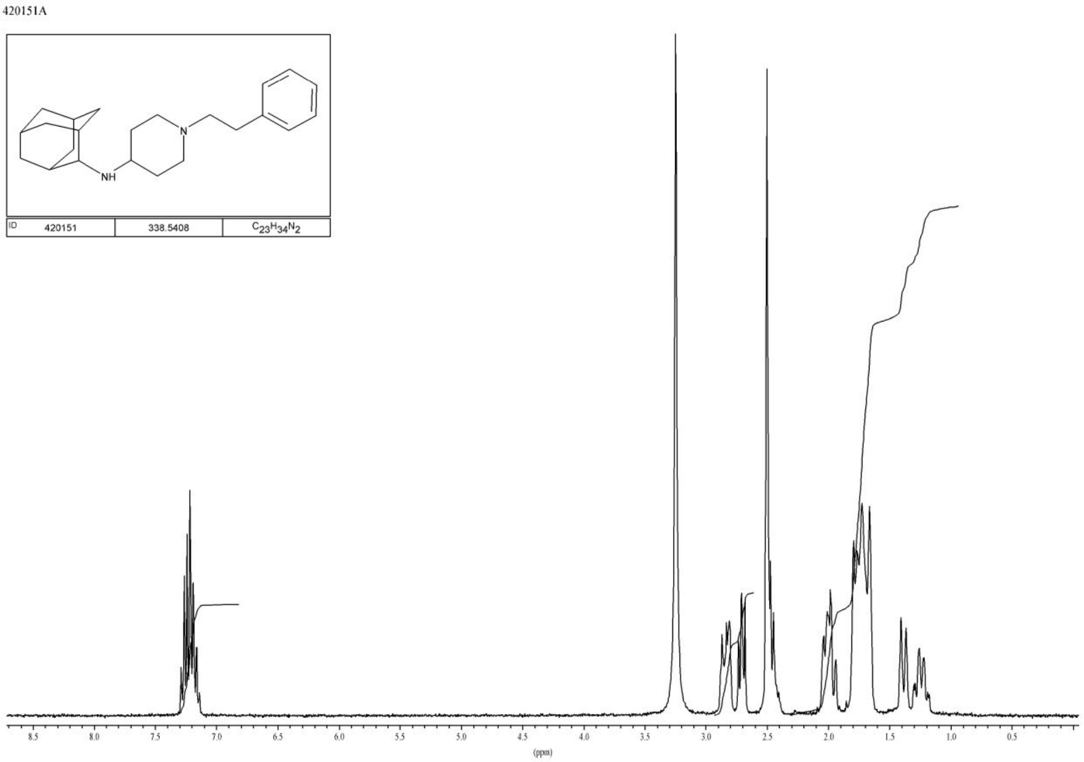

LDT-702

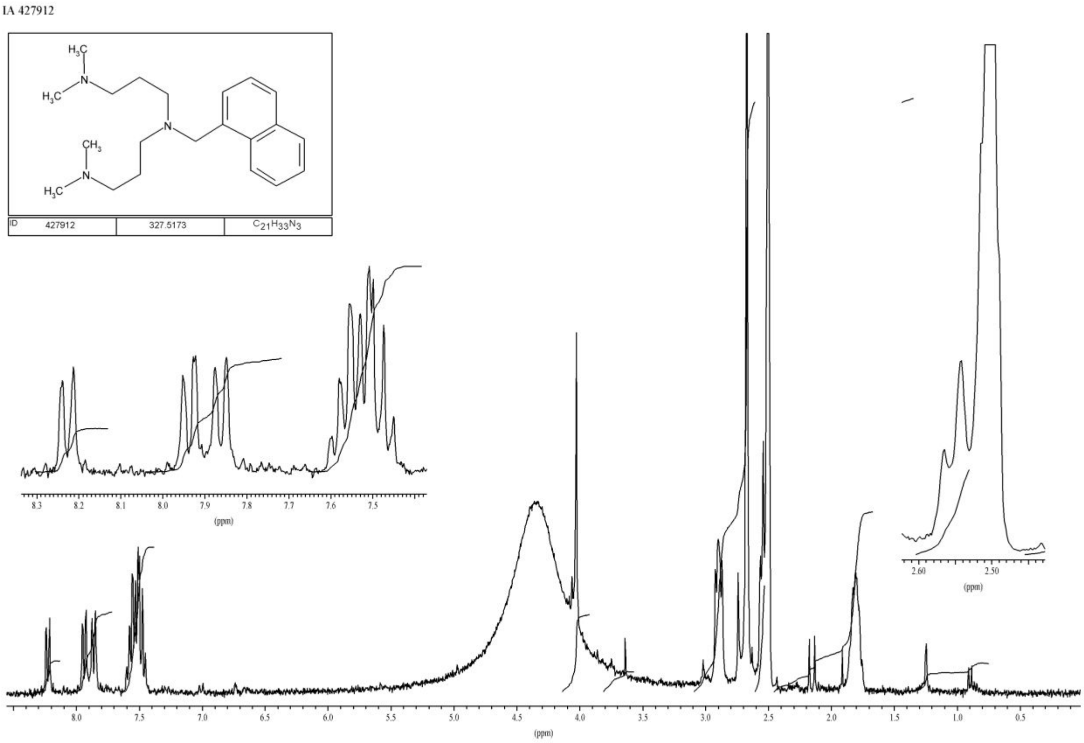

LDT-703

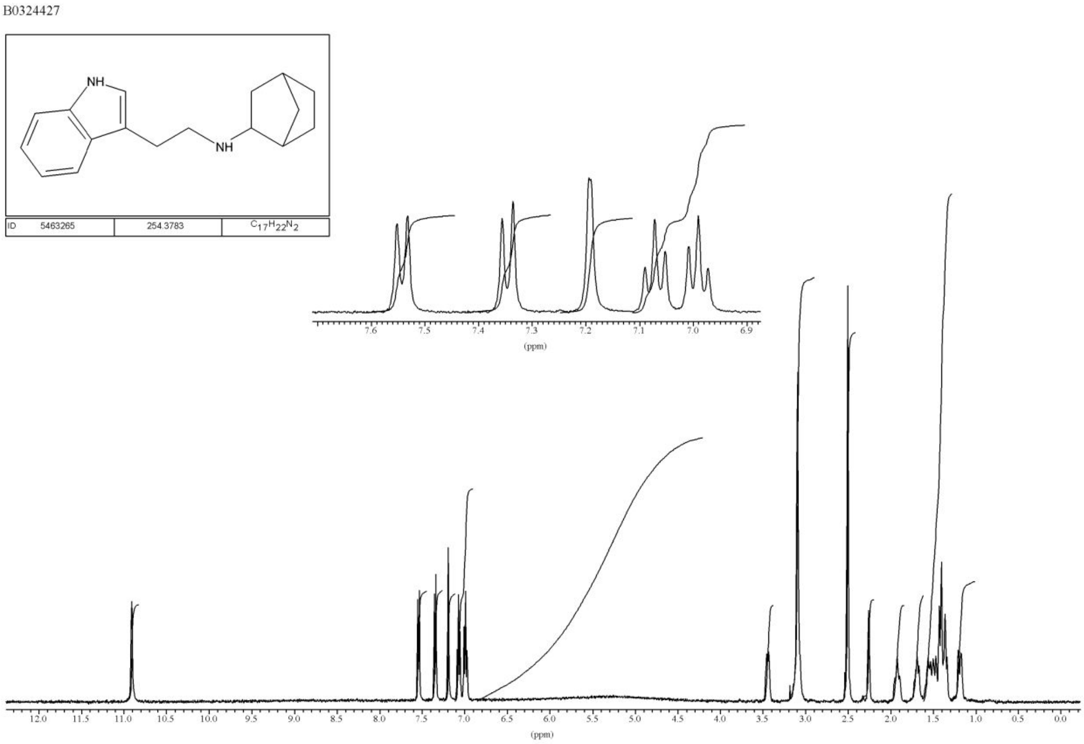

LDT-704

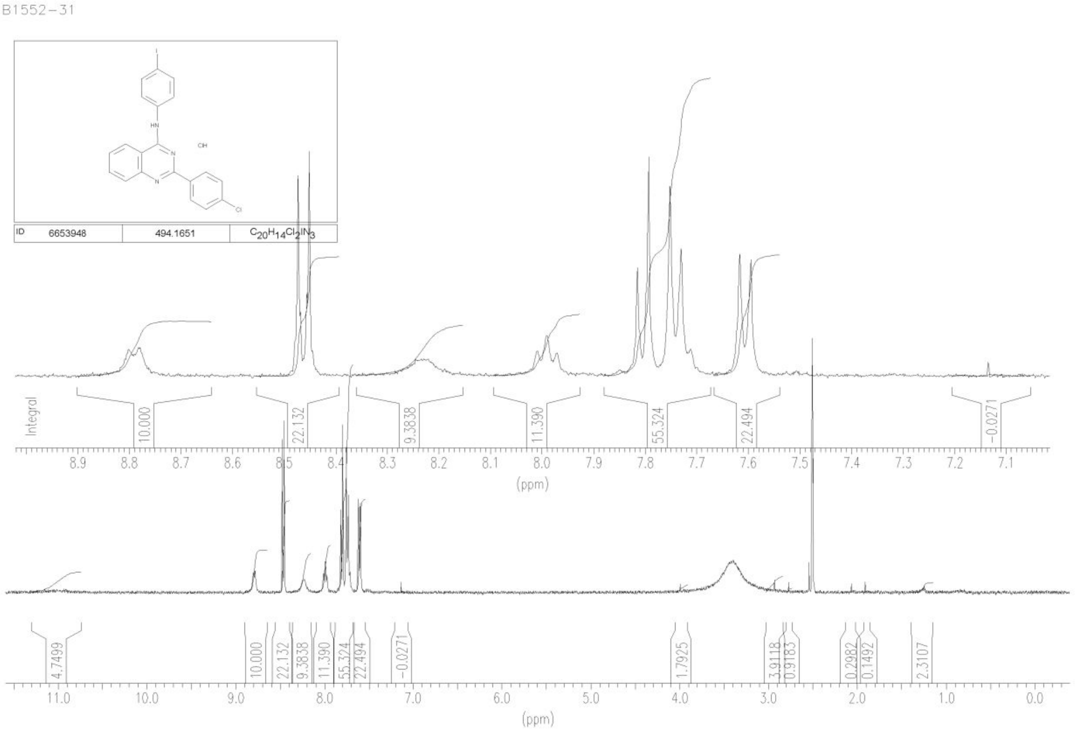

LDT-705

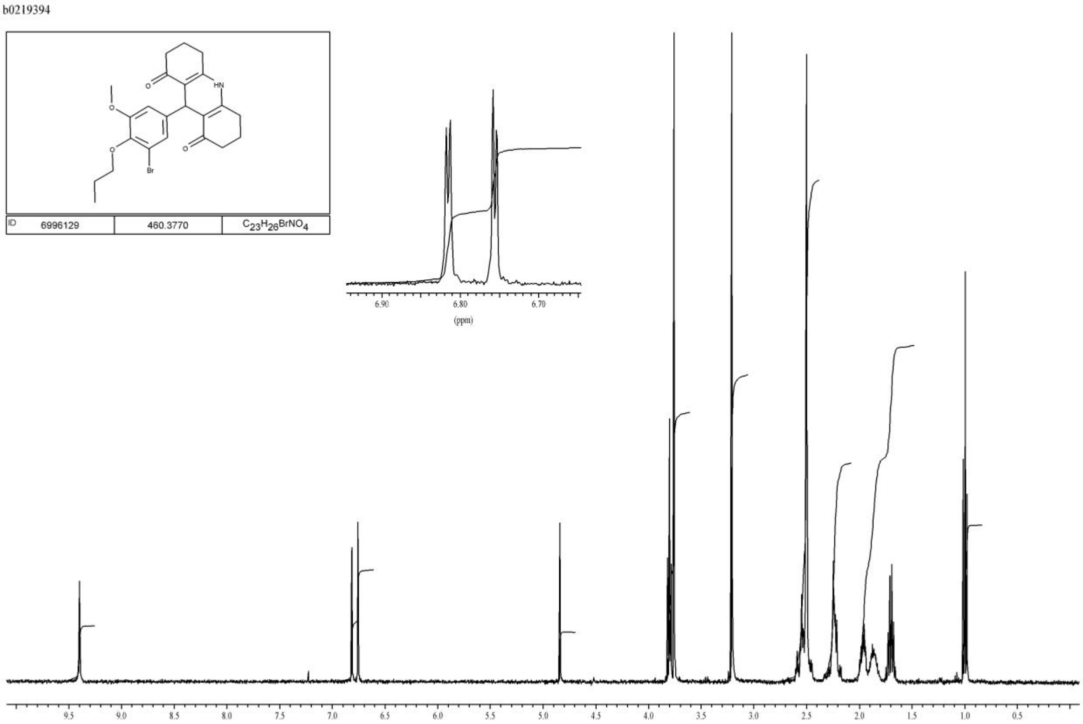

LDT-706

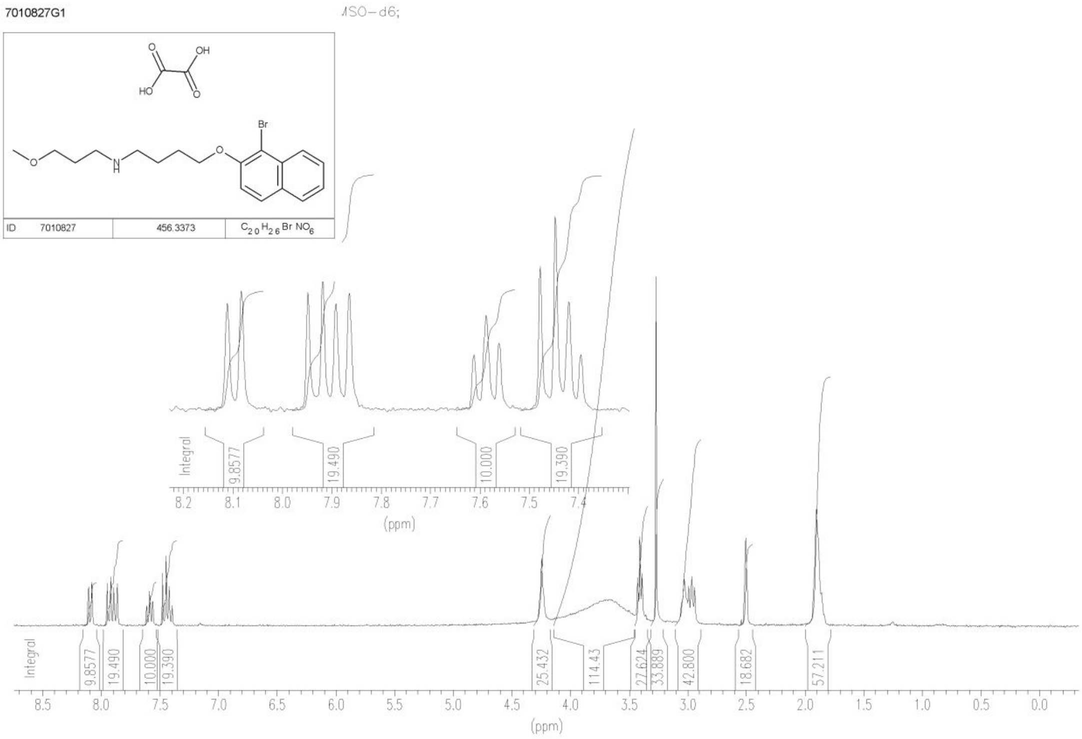

7010827G1
MSO-d6;
Br
OH
HO
O
N
H
ID
7010827
456.3373
$C_{20}H_{26}BrNO_6$
Integral
9.8577
19.490
10.000
19.390
(ppm)
25.432
114.43
27.624
33.889
42.800
18.682
57.211

LDT-707

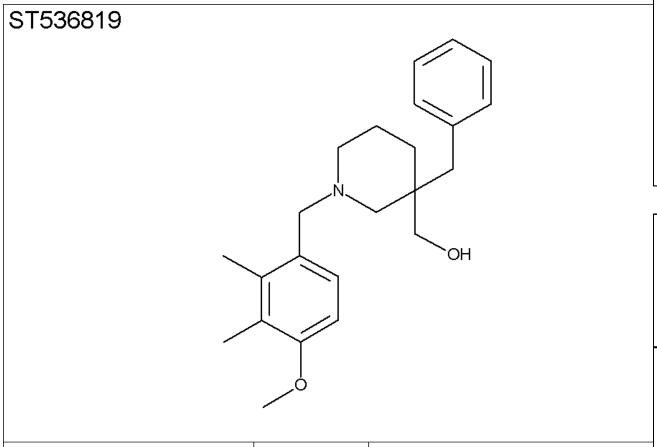

ID	18766896	353.5090	$C_{23}H_{31}NO_2$

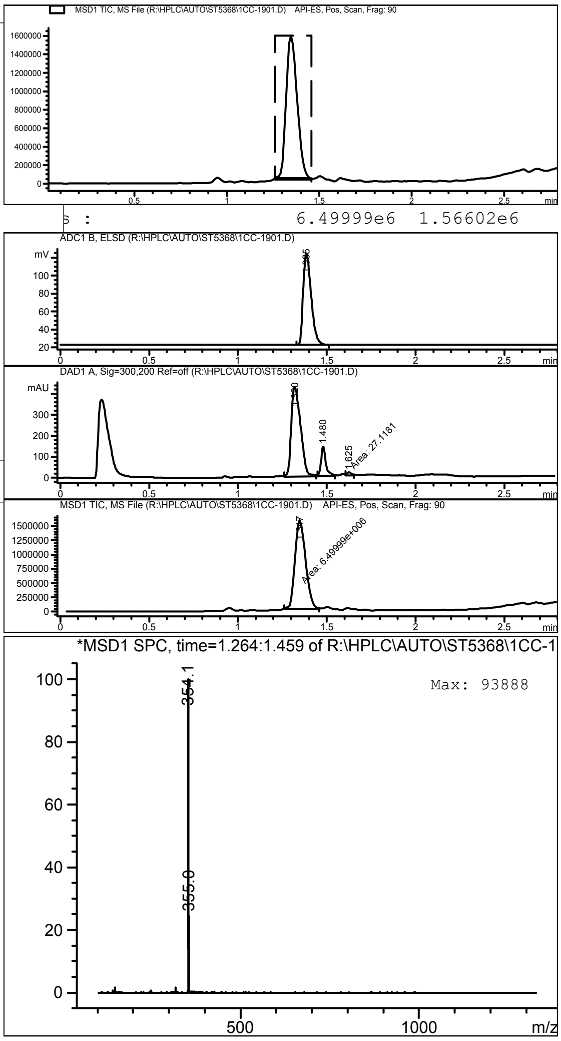


```
Data File R:\HPLC\AUTO\ST5368\1CC-1901.D
Sample Name: ST5368P1-C-03
Instrument 1 05/04/2019 10:40:17
Column: Onyx C18 50x4.6mm | 3.75ml/min | Columns
Reg Valve
Gradient: "A"->@2.0min->"B"(Hold 0.6min)-
>@0.2min->"A"->PostRun
PMP1, Solvent A         : 0.1%TFA, 2.5%AcN in H2O
PMP1, Solvent B         : 0.1%TFA in AcN
PMP1, Solvent C         : --NOT USED--
PMP1, Solvent D         : --NOT USED--
Ionization mode              :    API-ES Positive

Signal 1: ADC1 B, ELSD
Peak RetTime Type  Width     Area      Height
Area
  #   [min]         [min]   [mV*s]      [mV]
%
----|-------|----|-------|----------|----------|
   1   1.385 PB    0.0495  318.28976  101.78110
100.0000
Totals : 318.28976  101.78110

Signal 2: DAD1 A, Sig=300,200 Ref=off
Peak RetTime Type  Width     Area      Height
Area
  #   [min]         [min]   [mAU*s]     [mAU]
%
----|-------|----|-------|----------|----------|
   1   1.320 BB    0.0607 1563.14319  426.75388
83.9309
   2   1.480 BB    0.0286  272.15598  140.61423
14.6131
   3   1.625 MM    0.0293   27.11809   15.45054
1.4561
Totals :                  1862.41725  582.81864

Signal 3: MSD1 TIC, MS File
Peak RetTime Type  Width     Area      Height
Area
  #   [min]         [min]
%
----|-------|----|-------|----------|----------|
1   1.347 MM    0.0692 6.49999e6  1.56602e6
100.0000
```

LDT-708

FC94492647

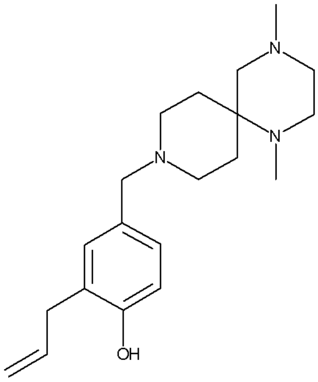

ID	41481383	329.4896	$C_{20}H_{31}N_3O$

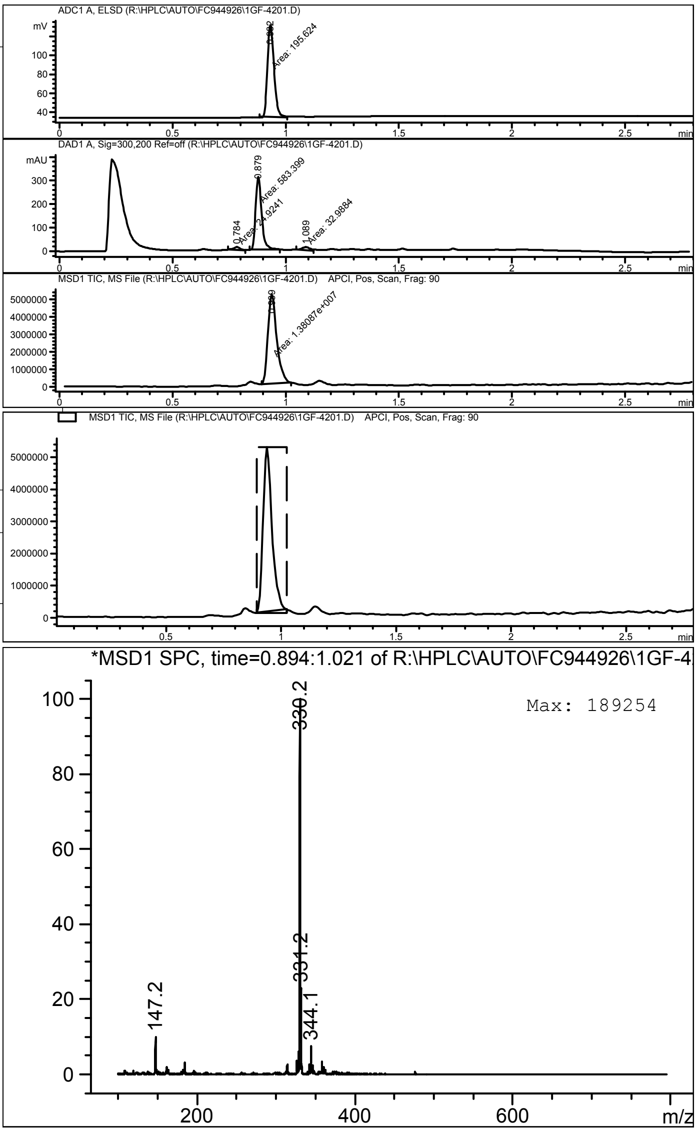

Data File R:\HPLC\AUTO\FC944926\1GF-4201.D
Sample Name: Fc944926P1-G-06
Instrument 1 09/08/2011 10:39:27
PMP1, Solvent A : 0.1%TFA in Acn/H2O (2.5:97.5)
PMP1, Solvent B : 0.1% TFA in AcN
PMP1, Solvent C : 0.1%FA in ACN/H2O (2.5:97.5)
PMP1, Solvent D : 0.1%FA in ACN
Ionization mode : APCI Positive

Signal 1: ADC1 A, ELSD

Peak #	RetTime [min]	Type	Width [min]	Area [mV*s]	Height [mV]	Area %
1	0.932	MM	0.0337	195.62422	96.76506	100.0000
Totals :				195.62422	96.76506	

Signal 2: DAD1 A, Sig=300,200 Ref=off

Peak #	RetTime [min]	Type	Width [min]	Area [mAU*s]	Height [mAU]	Area %
1	0.784	MM	0.0337	24.92410	12.32277	3.8864
2	0.879	MM	0.0313	583.39941	310.83698	90.9697
3	1.089	MM	0.0408	32.98842	13.48815	5.1439
Totals :				641.31194	336.64790	

Signal 3: MSD1 TIC, MS File

Peak #	RetTime [min]	Type	Width [min]	Area	Height	Area %
1	0.939	MM	0.0447	1.38086e7	5.15302e6	100.0000
Totals :				1.38087e7	5.15302e6	

LDT-709

FC943587727

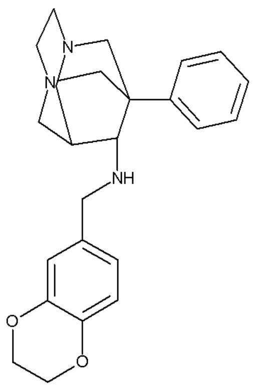

ID	46294896	391.5176	$C_{24}H_{29}N_3O_2$

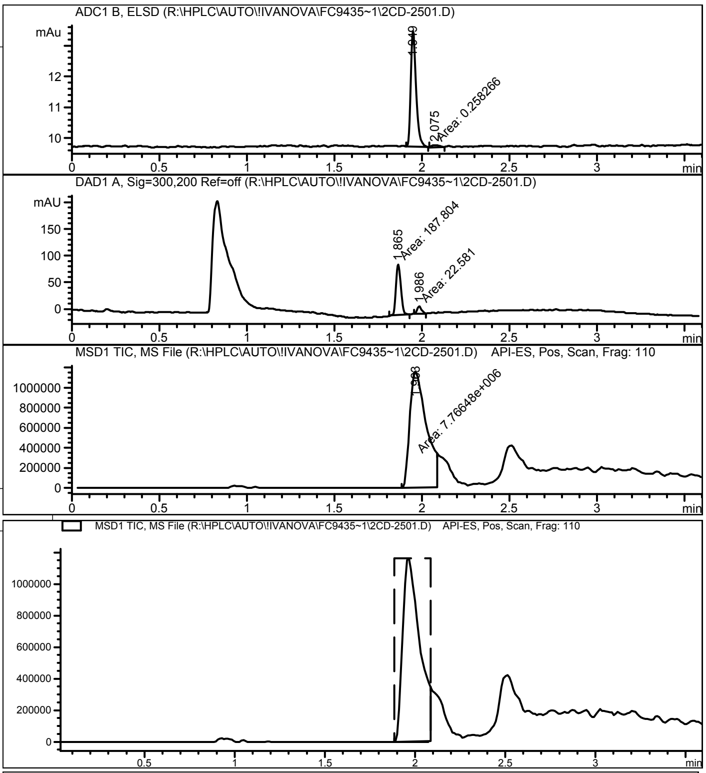

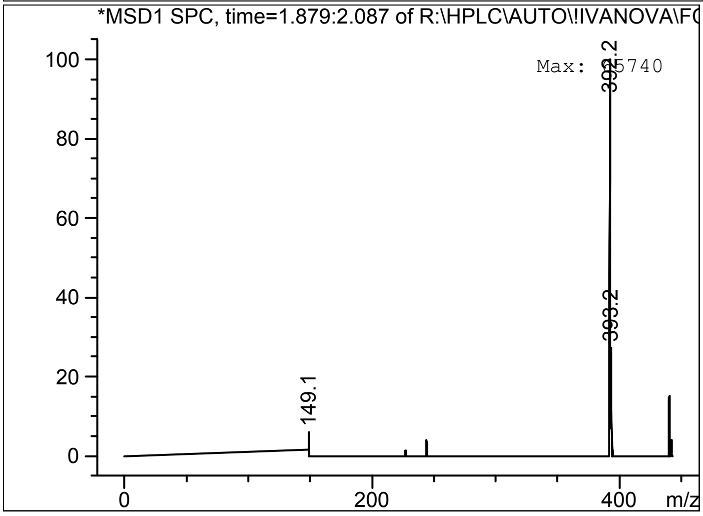

Data File R:\HPLC\AUTO\!IVANOVA\FC9435~1\2CD-2501.D
Sample Name: FC9435877P2-C-04
Instrument 1 15/09/2023 9:39:43 PM
Injection Date : 14/09/2023 16:40:2
Column: C18 50x2.1mm | 0.80ml/min | Columns Reg Valve
Gradient: "A"->@2.2min->"B"(Hold 0.4min)->@0.01min->"A"(Hold 1.0min)->PostRun
PMP1, Solvent A : 0.1%TFA in Acn/H2O (2.5:97.5)
PMP1, Solvent B : 0.1%TFA in AcN
PMP1, Solvent C : 0.1%FA in Acn/H2O (2.5:97.5)
PMP1, Solvent D : 0.1%FA in AcN
Ionization mode : API-ES Positive

Signal 1: ADC1 B, ELSD

Peak #	RetTime [min]	Type	Width [min]	Area [mAu*s]	Height [mAu]	Area %
1	1.949	PP	0.0324	8.17105	3.74848	96.9361
2	2.075	MM	0.0531	2.58266e-1	8.10745e-2	3.0639
Totals :				8.42931	3.82956	

Signal 2: DAD1 A, Sig=300,200 Ref=off

Peak #	RetTime [min]	Type	Width [min]	Area [mAU*s]	Height [mAU]	Area %
1	1.865	MM	0.0329	187.80368	95.14594	89.2668
2	1.986	MM	0.0280	22.58095	13.44897	10.7332
Totals :				210.38463	108.59490	

Signal 3: MSD1 TIC, MS File

Peak #	RetTime [min]	Type	Width [min]	Area	Height	Area %
1	1.963	MM	0.1104	7.76647e6	1.17209e6	100.0000
Totals :				7.76648e6	1.17209e6	

References

(1) Kim, S.; Chen, J.; Cheng, T.; Gindulyte, A.; He, J.; He, S.; Li, Q.; Shoemaker, B. A.; Thiessen, P. A.; Yu, B.; et al. PubChem in 2021: New Data Content and Improved Web Interfaces. *Nucleic Acids Res.* **2021**, *49* (D1), D1388–D1395. https://doi.org/10.1093/nar/gkaa971.

(2) Gaulton, A.; Hersey, A.; Nowotka, M.; Bento, A. P.; Chambers, J.; Mendez, D.; Mutowo, P.; Atkinson, F.; Bellis, L. J.; Cibrián-Uhalte, E.; et al. The ChEMBL Database in 2017. *Nucleic Acids Res.* **2017**, *45* (D1), D945–D954. https://doi.org/10.1093/nar/gkw1074.

(3) Gamo, F. J.; Sanz, L. M.; Vidal, J.; De Cozar, C.; Alvarez, E.; Lavandera, J. L.; Vanderwall, D. E.; Green, D. V. S.; Kumar, V.; Hasan, S.; et al. Thousands of Chemical Starting Points for Antimalarial Lead Identification. *Nature* **2010**, *465* (7296), 305–310. https://doi.org/10.1038/nature09107.

(4) Delves, M. J.; Miguel-Blanco, C.; Matthews, H.; Molina, I.; Ruecker, A.; Yahiya, S.; Straschil, U.; Abraham, M.; León, M. L.; Fischer, O. J.; et al. A High Throughput Screen for Next-Generation Leads Targeting Malaria Parasite Transmission. *Nat. Commun.* **2018**, *9* (1), 3805. https://doi.org/10.1038/s41467-018-05777-2.

(5) Duffy, S.; Avery, V. M. Identification of Inhibitors of Plasmodium Falciparum Gametocyte Development. *Malar. J.* **2013**, *12* (1), 408. https://doi.org/10.1186/1475-2875-12-408.

(6) Antonova-Koch, Y.; Meister, S.; Abraham, M.; Luth, M. R.; Ottilie, S.; Lukens, A. K.; Sakata-Kato, T.; Vanaerschot, M.; Owen, E.; Jado, J. C.; et al. Open-Source Discovery of Chemical Leads for next-Generation Chemoprotective Antimalarials. *Science (80-.).* **2018**, *362* (6419), eaat9446. https://doi.org/10.1126/science.aat9446.

(7) Maaten, L. van der; Hinton, G. Visualizing Data Using T-SNE. *J. Mach. Learn. Res.* **2008**, *9*, 2579–2605.

(8) Morgan, H. L. The Generation of a Unique Machine Description for Chemical Structures-A Technique Developed at Chemical Abstracts Service. *J. Chem. Doc.* **1965**, *5* (2), 107–113. https://doi.org/10.1021/c160017a018.

(9) Landrum, G. RDKit: Open-Source Cheminformatics Software. 2017, p http://www.rdkit.org/.

(10) Tropsha, A. Best Practices for QSAR Model Development, Validation, and Exploitation. *Mol. Inform.* **2010**, *29* (6–7), 476–488. https://doi.org/10.1002/minf.201000061.

(11) Gobbi, A.; Poppinger, D. Genetic Optimization of Combinatorial Libraries. *Biotechnol. Bioeng.* **1998**, *61* (1), 47–54. https://doi.org/10.1002/(SICI)1097-0290(199824)61:1<47::AID-BIT9>3.0.CO;2-Z.

(12) Anderson, S. Graphical Representation of Molecules and Substructure-Search Queries in MACCStm. *J. Mol. Graph.* **1984**, *2* (3), 83–90. https://doi.org/10.1016/0263-7855(84)80060-0.

(13) Moriwaki, H.; Tian, Y.-S.; Kawashita, N.; Takagi, T. Mordred: A Molecular Descriptor Calculator. *J. Cheminform.* **2018**, *10* (1), 4. https://doi.org/10.1186/s13321-018-0258-y.

(14) Breiman, L. Random Forests. *Mach. Learn.* **2001**, *45*, 5–32. https://doi.org/10.1023/A:1010933404324.

(15) Pedregosa, F.; Varoquaux, G.; Gramfort, A.; Michel, V.; Thirion, B.; Grisel, O.; Blondel, M.; Prettenhofer, P.; Weiss, R.; Dubourg, V.; et al. Scikit-Learn: Machine Learning in Python. *J. Mach. Learn. Res.* **2012**, *12*, 2825–2830. https://doi.org/10.1007/s13398-014-0173-7.2.

(16) Wu, J.; Chen, X. Y.; Zhang, H.; Xiong, L. D.; Lei, H.; Deng, S. H. Hyperparameter Optimization for Machine Learning Models Based on Bayesian Optimization. *J. Electron. Sci. Technol.* **2019**, *17* (1), 26–40. https://doi.org/10.11989/JEST.1674-862X.80904120.

(17) Tropsha, A.; Golbraikh, A. Predictive QSAR Modeling Workflow, Model Applicability Domains, and Virtual Screening. *Curr. Pharm. Des.* **2007**, *13* (34), 3494–3504. https://doi.org/10.2174/138161207782794257.

(18) Sundararajan, M.; Najmi, A. The Many Shapley Values for Model Explanation. In *Proceedings of the 37th International Conference on Machine Learning Research, PMLR*; 2020; pp 9269–9278.

(19) Lundberg, S. M.; Erion, G.; Chen, H.; DeGrave, A.; Prutkin, J. M.; Nair, B.; Katz, R.; Himmelfarb, J.; Bansal, N.; Lee, S.-I. From Local Explanations to Global Understanding with Explainable AI for Trees. *Nat. Mach. Intell.* **2020**, *2* (1), 56–67. https://doi.org/10.1038/s42256-019-0138-9.

(20) Veber, D. F.; Johnson, S. R.; Cheng, H.-Y.; Smith, B. R.; Ward, K. W.; Kopple, K. D. Molecular Properties That Influence the Oral Bioavailability of Drug Candidates. *J. Med. Chem.* **2002**, *45* (12), 2615–2623. https://doi.org/10.1021/jm020017n.

(21) Lipinski, C. A.; Lombardo, F.; Dominy, B. W.; Feeney, P. J. Experimental and Computational Approaches to Estimate Solubility and Permeability in Drug Discovery and Development Settings. *Adv. Drug Deliv. Rev.* **1997**, *23* (1–3), 3–25. https://doi.org/10.1016/S0169-409X(96)00423-1.

(22) Trager, W.; Jensen, J. Human Malaria Parasites in Continuous Culture. *Science (80-.).* **1976**, *193* (4254), 673–675. https://doi.org/10.1126/science.781840.

(23) Smilkstein, M.; Sriwilaijaroen, N.; Kelly, J. X.; Wilairat, P.; Riscoe, M. Simple and Inexpensive Fluorescence-Based Technique for High-Throughput Antimalarial Drug Screening. *Antimicrob. Agents Chemother.* **2004**, *48* (5), 1803–1806. https://doi.org/10.1128/AAC.48.5.1803-1806.2004.

(24) Marin-Mogollon, C.; Salman, A. M.; Koolen, K. M. J.; Bolscher, J. M.; van Pul, F. J. A.; Miyazaki, S.; Imai, T.; Othman, A. S.; Ramesar, J.; van Gemert, G.-J.; et al. A P. Falciparum NF54 Reporter Line Expressing MCherry-Luciferase in Gametocytes, Sporozoites, and Liver-Stages. *Front. Cell. Infect. Microbiol.* **2019**, *9*, 96. https://doi.org/10.3389/fcimb.2019.00096.

(25) Fivelman, Q. L.; McRobert, L.; Sharp, S.; Taylor, C. J.; Saeed, M.; Swales, C. A.; Sutherland, C. J.; Baker, D. A. Improved Synchronous Production of Plasmodium Falciparum Gametocytes in Vitro. *Mol. Biochem. Parasitol.* **2007**, *154* (1), 119–123. https://doi.org/10.1016/j.molbiopara.2007.04.008.

(26) Calit, J.; Dobrescu, I.; Gaitán, X. A.; Borges, M. H.; Ramos, M. S.; Eastman, R. T.; Bargieri, D. Y. Screening the Pathogen Box for Molecules Active against Plasmodium Sexual Stages Using a New Nanoluciferase-Based Transgenic Line of P. Berghei Identifies Transmission-Blocking Compounds. *Antimicrob. Agents Chemother.* **2018**, *62* (11). https://doi.org/10.1128/AAC.01053-18.

(27) Blagborough, A. M.; Delves, M. J.; Ramakrishnan, C.; Lal, K.; Butcher, G.; Sinden, R. E. Assessing Transmission Blockade in Plasmodium Spp. In *Methods in Molecular Biology (Methods and Protocols)*; Ménard, R., Ed.; Humana Press: Totowa, NJ, 2012; pp 577–600. https://doi.org/10.1007/978-1-62703-026-7_40.

(28) Mendes, A. M.; Albuquerque, I. S.; Machado, M.; Pissarra, J.; Meireles, P.; Prudêncio, M. Inhibition of Plasmodium Liver Infection by Ivermectin. *Antimicrob. Agents Chemother.* **2017**, *61* (2). https://doi.org/10.1128/AAC.02005-16.
